# Surgery After Induction Therapy for Cervical Esophageal Cancer: A Systematic Review and Proposed Multidisciplinary Selection Framework

**DOI:** 10.3390/cancers18111736

**Published:** 2026-05-26

**Authors:** Ismaell Massalha, Adham Hijab, Reem Zabit, Bilal Krayim, Wael Hozaeel, Moatz Safadi, Samer Hussany, Israel Sandler, Jamal Zidan, Ofir Cohen, Ory Wiesel

**Affiliations:** 1 Department of Radiation Oncology, Ziv Medical Center, Safed 1300000, Israel; 2 Faculty of Health Sciences, Ben-Gurion University, Beer Sheva 8410501, Israel; 3 Department of Pediatrics, Ziv Medical Center, Safed 1300000, Israel; 4 Helmsley Cancer Center, Shaare Zedek Medical Center, Jerusalem 9103102, Israel; 5 Legacy Heritage Oncology Center & Dr. Larry Norton Institute, Soroka University Medical Center, Beer Sheva 8410101, Israel; 6 Department of Oncology, Ziv Medical Center, Safed 1300000, Israel; 7 Department of Nuclear Medicine, Ziv Medical Center, Safed 1300000, Israel; 8 Faculty of Computer and Information Science, Ben-Gurion University of the Negev, Beer Sheva 8410501, Israel; 9 Baruch Padeh-Tzafon Medical Center, Poriya 1528001, Israel

**Keywords:** cervical esophageal cancer, neoadjuvant chemoimmunotherapy, esophagectomy, larynx preservation, patient selection framework, pathologic complete response, adjuvant nivolumab, surgical timing, PRISMA systematic review

## Abstract

**Background/Objectives:** Management of cervical esophageal cancer after induction therapy remains unsettled. Definitive chemoradiotherapy is the guideline default, but a subset of patients with residual but resectable disease may still benefit from surgery. No validated multidisciplinary selection framework exists for this subsite. **Methods:** We conducted a systematic review registered in the International Prospective Register of Systematic Reviews (PROSPERO; CRD420261369102) and guided by the Preferred Reporting Items for Systematic Reviews and Meta-Analyses (PRISMA) 2020 statement, using searches of PubMed/MEDLINE, Web of Science, Scopus, and the Cochrane Library from inception through 14 April 2026. We identified 1779 records, removed 873 duplicates, and screened 906 records; 87 full-text reports were assessed, of which 67 were excluded at the full-text stage (66 on population grounds—disease not cervical esophageal; and 1 because cervical-direct outcomes were not separable within a mixed cervical/thoracic cohort), leaving 20 cervical-direct studies included in the primary synthesis. Thoracic and meta-analytic sources are cited for indirect comparison and biological rationale but are not counted in the included set. Included studies were evaluated using the Newcastle–Ottawa Scale (NOS) and Risk Of Bias In Non-randomised Studies of Interventions (ROBINS-I); certainty of evidence was assessed using the Grading of Recommendations, Assessment, Development and Evaluation (GRADE) framework. Formal meta-analysis was not performed because study design, treatment approach, and outcome reporting were too heterogeneous. **Results:** Cervical-specific evidence is predominantly retrospective but consistent in direction. Available cervical-specific observational data suggest benefit mainly in patients with biopsy-confirmed incomplete response, resectable residual disease, preserved performance status, and access to experienced centers. Larynx-preserving resection is feasible in 90% of T1–2 tumors and 54% of T3–4 responders. In thoracic esophageal squamous cell carcinoma, neoadjuvant chemoimmunotherapy yields pathologic complete response rates of approximately 29–48%; in cervical disease, the SCENIC trial has reported interim clinical response of approximately 50% in 28 patients, but pathology-confirmed response is not yet available. We present a proposed multidisciplinary selection framework integrating response depth, post-induction stage, laryngeal preservation feasibility, sarcopenia, circulating tumor DNA dynamics, and programmed death-ligand 1 (PD-L1) expression. The framework has not been prospectively validated and is presented as a hypothesis-generating, conceptual tool for multidisciplinary discussion rather than a clinically validated instrument. Adjuvant nivolumab is recommended for residual pathologic disease after margin-negative (R0) resection when surgery follows preoperative chemoradiotherapy; after PD-1-based induction, adjuvant checkpoint inhibition remains investigational. **Conclusions:** The available cervical-direct evidence is predominantly retrospective and selection-prone, and several inputs supporting the framework are extrapolated from thoracic ESCC cohorts; conclusions about the survival benefit of surgery should therefore be read as associations rather than causal claims. Surgery has a role after induction therapy in carefully selected incomplete responders. The proposed framework is designed for multidisciplinary use and requires prospective validation before routine clinical application.

## Simple Summary

Cervical esophageal cancer is rare, anatomically difficult, and usually treated with definitive chemoradiotherapy. Some patients with residual but resectable disease after induction therapy may benefit from surgery, but selection remains poorly defined. We identified 1779 records and, after deduplication and screening, assessed 87 full-text reports; 20 cervical-direct studies met the primary inclusion criterion and form the synthesis. Thoracic and meta-analytic sources are cited throughout for indirect comparison and biological rationale but are not counted as included studies. The evidence supports surgery only for selected incomplete responders with adequate fitness and technically feasible resection. We propose a multidisciplinary framework integrating response, stage, larynx-preservation feasibility, physiologic reserve, sarcopenia, and selected biomarkers. The framework is for multidisciplinary discussion and requires prospective validation before routine clinical use.

## 1. Introduction

Cervical esophageal cancer (CEC) arises between the cricopharyngeus muscle and the thoracic inlet, corresponding to the C6–T1 vertebral level [1,2]. It accounts for 2–10% of all esophageal malignancies, with esophageal squamous cell carcinoma (ESCC) comprising the majority [2,3]. The anatomical position imposes constraints that distinguish cervical from thoracic disease: proximity to the larynx, trachea, great vessels, and recurrent laryngeal nerves restricts surgical access and substantially increases the risk of voice and swallowing impairment with any intervention [1,4,5]. Patients usually present with progressive dysphagia and unintended weight loss, often to an otolaryngology or general surgery service, and the diagnostic workup—cross-sectional imaging, upper endoscopy with biopsy, and PET/CT for staging—should be completed before any treatment decision is made within a multidisciplinary committee that also addresses pretreatment nutritional support [2,6].

Historically, surgery required total pharyngolaryngoesophagectomy with permanent tracheostomy, and the functional toll of this approach drove adoption of definitive chemoradiotherapy as the primary treatment modality [1,7]. Current guidelines reflect this history. The National Comprehensive Cancer Network (NCCN) recommends definitive chemoradiotherapy as the standard approach for cervical or cervicothoracic esophageal squamous cell carcinoma arising within 5 cm of the cricopharyngeus; esophagectomy is specified for non-cervical disease [8]. The 2024 Lancetreview similarly identifies definitive chemoradiotherapy as the primary option for cervical tumors and notes that surgery may improve prognosis for selected T3–T4a disease after induction [6]. Definitive chemoradiotherapy carries its own long-term costs, however: stricture formation, chronic dysphagia, and limited salvage options when local disease recurs [2,6].

The integration of immune checkpoint inhibitors into neoadjuvant regimens has altered the treatment landscape for thoracic esophageal squamous cell carcinoma, with pathologic complete response rates of 29–48% reported with chemoimmunotherapy, substantially higher than the 15–20% historically associated with chemotherapy alone [9,10,11,12]. Whether these response rates are achievable at the cervical level, and whether they translate into a meaningful survival benefit given the anatomic and functional constraints of this subsite, remains unanswered. The Stratified Treatment of Localized Cervical Esophageal Squamous Cell Carcinoma Induced by Neoadjuvant Immunotherapy Plus Chemotherapy (SCENIC) trial provides the only prospective cervical-specific chemoimmunotherapy data, reporting approximately 50% clinical response at interim analysis in 28 patients treated with tislelizumab plus nab-paclitaxel and carboplatin, with grade ≥3 adverse events in 16% [13]; long-term outcomes remain unavailable.

After induction, the practical question is not simply whether residual disease can be removed. It is whether surgery is likely to help the patient in front of the MDT. That remains one of the least settled problems in upper gastrointestinal oncology. Throughout this review, cervical-specific studies were prioritized for treatment selection, surgical feasibility, and functional outcome assessment. Thoracic data were used only when clinically informative, and are labeled as extrapolative. We synthesize the available evidence, propose a structured multidisciplinary selection framework for surgical candidacy after induction therapy, and define the evidence gaps that need prospective testing.

## 2. Methods

### 2.1. Study Design

This study was conducted as a systematic review with structured narrative synthesis to evaluate the role of surgery following induction therapy for cervical esophageal cancer. The review addressed response-adapted treatment strategies, the evolving role of chemoimmunotherapy-based induction regimens, larynx-preserving surgical approaches, and oncologic and functional outcomes. Because of substantial heterogeneity in study design, patient selection, tumor location, treatment protocols, and outcome reporting, quantitative meta-analysis was not performed.

The review was conducted and reported according to the Preferred Reporting Items for Systematic Reviews and Meta-Analyses (PRISMA) 2020 statement, including predefined eligibility criteria, dual-stage study selection, and explicit reporting of exclusion reasons. The review is registered in PROSPERO (CRD420261369102; registration date 14 April 2026).

### 2.2. Search Strategy

A systematic search of the literature was conducted in PubMed/MEDLINE, Web of Science, Scopus, and the Cochrane Library from database inception through 14 April 2026, which served as the final search round for this review. Reference lists of included studies and relevant reviews were also manually screened to identify additional eligible reports.

The search strategy combined controlled vocabulary and free-text terms related to cervical esophageal malignancy, induction treatment, surgical management, and immunotherapy. Core terms included “cervical esophageal cancer”, “cervical esophageal carcinoma”, “proximal esophageal cancer”, “esophageal squamous cell carcinoma”, “induction therapy”, “neoadjuvant therapy”, “chemoradiotherapy”, “chemoimmunotherapy”, “immune checkpoint inhibitor”, “PD-1”, “PD-L1”, “esophagectomy”, “larynx-preserving surgery”, “pharyngolaryngoesophagectomy”, “salvage surgery”, and “conversion surgery”. Boolean operators (AND/OR) were applied to refine retrieval.

The search strategy was intentionally broad in order to maximize sensitivity, given the rarity of cervical esophageal cancer and the limited number of cervical-specific contemporary studies. Full search strategies for all databases are provided in Supplementary Materials Table S1.

### 2.3. Eligibility Criteria

Studies were considered eligible if they: (i) included patients with cervical esophageal cancer, or reported extractable cervical subgroup data within broader esophageal squamous cell carcinoma cohorts; (ii) evaluated induction or neoadjuvant treatment followed by surgery; (iii) reported oncologic, surgical, or functional outcomes relevant to the study objectives; and (iv) were designed as prospective clinical trials, retrospective cohort studies, registry-based analyses, or clinically informative surgical case series.

Because prospective cervical-specific evidence remains limited, selected studies in thoracic esophageal squamous cell carcinoma were also considered when they were clinically relevant to induction strategies, response-adapted treatment allocation, or surgical decision making, provided that their applicability to cervical disease could be reasonably interpreted. Such studies were used cautiously and are identified as extrapolative where appropriate.

Exclusion criteria included non-relevant histology without separable squamous-cell-specific data, non-surgical treatment-only series, studies without extractable outcomes of interest, preclinical studies, editorials, commentaries, narrative reviews without primary data, and duplicate or overlapping cohorts when a more complete dataset was available.

### 2.4. Study Selection

Title and abstract screening was performed independently by two reviewers (I.M. and A.H.); full-text eligibility was assessed independently by the same two reviewers. Disagreements at each stage were resolved by consensus with a third reviewer (O.W.).

**Figure 1:**
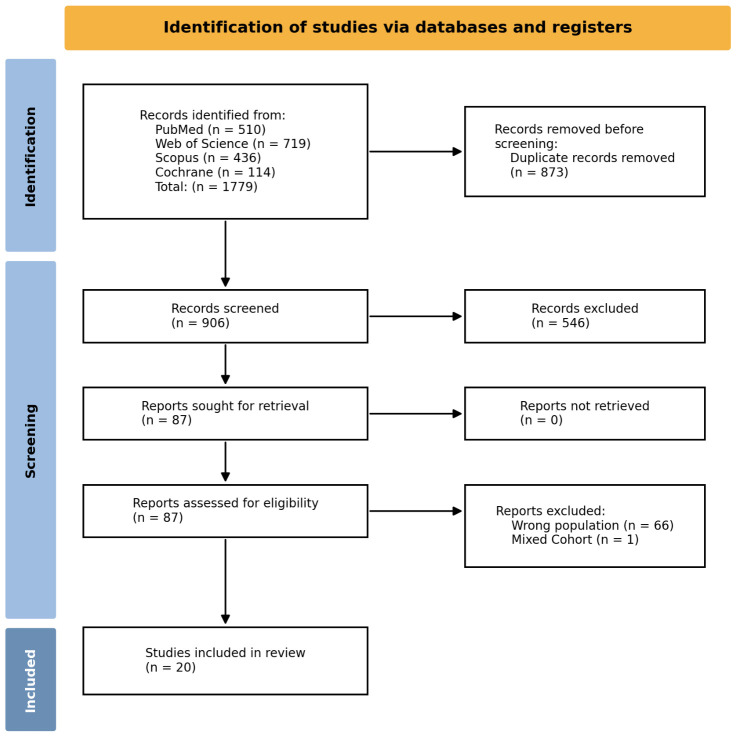
PRISMA 2020 flow diagram of study selection.

Across the four databases, 1779 records were identified. After removal of 873 duplicates, 906 unique records underwent title and abstract screening. Records excluded at title and abstract stage and reports not retrievable (predominantly conference abstracts or records without accessible full-text articles) were logged in the PRISMA flow diagram; 87 reports were assessed at full-text stage. Sixty-seven reports were excluded at the full-text stage: 66 on population grounds (disease not cervical esophageal) and 1 on mixed-cohort grounds (cervical-direct outcomes not separable within a combined cervical/thoracic population). Twenty cervical-direct studies met the primary inclusion criterion and form the synthesis. Thoracic and meta-analytic evidence, including the single phase 3 randomized trial in ESCC (ESCORT-NEO; Qin 2024), is retained as supporting evidence and cited throughout the Methods and Results for indirect comparison and biological rationale; it is not counted in the included set and is not subject to the NOS/ROBINS-I quality assessment reported for the primary synthesis. The PRISMA flow diagram (Figure 1) summarizes the numeric counts; the full list of studies excluded at full-text stage, with reasons, is provided in Supplementary Materials Table S2. Table 1 presents the primary-included cervical-direct studies (sections A, B, and E) and, for context, the principal thoracic and meta-analytic sources cited in the review (sections C and D). Additional supporting evidence used for indirect comparison is compiled in Supplementary Materials Tables S3 and S4. A descriptive synthesis of the cervical-direct primary-included studies is provided in Table 2; the proposed multidisciplinary selection framework is presented in Table 3 and Table 4; major perioperative complications are compared in Table 5.

### 2.5. Data Extraction

Data extraction was performed independently by two reviewers (I.M. and A.H.) using a predefined structured template; discrepancies were resolved by discussion and, where necessary, adjudicated by a third reviewer (O.W.). Extracted variables included study characteristics (author, year, country, design, sample size), population characteristics, tumor location, induction regimen, radiotherapy and systemic treatment details, surgical approach, laryngeal preservation strategy, pathological response data, perioperative outcomes, and oncologic endpoints including resection status, recurrence patterns, and survival outcomes.

“NR” (not reported) was used for variables absent from the original source. When numerical survival estimates were not directly reported, outcome data were interpreted from published Kaplan–Meier curves where feasible; such approximations are explicitly identified where used.

Risk of bias for retrospective cohort and registry-based comparative studies was assessed using the Newcastle–Ottawa Scale (NOS); risk of bias for prospective non-randomized interventional studies was assessed using the ROBINS-I tool; and risk of bias for the single phase 3 randomized trial in ESCC informing indirect evidence (ESCORT-NEO/NCCES01 [14]) was assessed using the Cochrane Risk of Bias 2 (RoB 2) tool. Assessments were performed independently by two reviewers (I.M. and A.H.) with discrepancies resolved by consensus; results are reported in Supplementary Materials Table S5 (NOS), Supplementary Materials Table S6 (ROBINS-I), and Supplementary Materials Table S7 (RoB 2). Risk-of-bias findings informed the weight placed on each study during narrative interpretation but were not used as exclusion criteria, given the predominantly retrospective, observational evidence base. Certainty of evidence for key outcomes was assessed using the GRADE framework (Supplementary Materials Table S8).

Given the heterogeneity of study populations, anatomical subsites, induction regimens, operative strategies, and reported endpoints, findings were synthesized qualitatively rather than pooled quantitatively.

The synthesis was organized around four prespecified clinical questions: (1) which patients may benefit from surgery after induction therapy; (2) how chemoimmunotherapy may influence response, resectability, and postoperative outcomes; (3) when larynx-preserving surgery is feasible; and (4) where evidence remains insufficient to support firm clinical recommendations.

Cervical-specific evidence was prioritized throughout. When cervical data were sparse, supportive data from thoracic esophageal squamous cell carcinoma studies, national datasets, and meta-analyses including cervical cases were incorporated with explicit acknowledgment of anatomical and functional differences.

To keep direct and indirect evidence distinct, we used a prespecified evidence-to-decision hierarchy for narrative interpretation. Cervical-direct studies were treated as the primary evidence base and carried full interpretive weight. Mixed cervicothoracic or registry studies with extractable cervical estimates were treated as supportive cervical evidence. Thoracic-only trials, thoracic-dominant meta-analyses, and biomarker studies were discounted for indirectness and used mainly to anchor biologic rationale, response benchmarks, or postoperative management where cervical data were absent. This hierarchy was cross-walked to the GRADE indirectness judgments reported in Supplementary Materials Table S8 and summarized in Supplementary Materials Table S9. The weights are consensus- and evidence-informed rather than statistically optimized.

## 3. The Evolving Role of Chemoimmunotherapy in Induction Treatment

### 3.1. Rationale for Immunotherapy in Esophageal Squamous Cell Carcinoma

Esophageal squamous cell carcinoma exhibits high tumor mutational burden, frequent programmed death-ligand 1 (PD-L1) expression, and an immunogenic tumor microenvironment, providing a biological rationale for immune checkpoint inhibitor therapy [6]. In the metastatic setting, pembrolizumab and nivolumab have demonstrated survival benefits in PD-L1-positive tumors, leading to regulatory approval [8]. These results prompted investigation of neoadjuvant chemoimmunotherapy in resectable disease.

### 3.2. Neoadjuvant Chemoimmunotherapy: Evidence from Thoracic Esophageal Squamous Cell Carcinoma

Multiple retrospective and prospective studies have evaluated neoadjuvant chemoimmunotherapy in predominantly thoracic esophageal squamous cell carcinoma. Pathologic complete response rates range from approximately 29% to 48%, substantially higher than historical chemotherapy-alone regimens (15–20%) [9,10,11,12]. The SCENIC trial, a prospective phase II study in cervical esophageal cancer specifically, reported neoadjuvant tislelizumab plus nab-paclitaxel and carboplatin in 28 patients, with grade ≥3 adverse events in 16% and an interim clinical response signal [13]. This cervical signal is clinical rather than pathology-confirmed, comes from a small interim cohort, and should not be quantitatively compared with thoracic pCR benchmarks.

A 2025 meta-analysis of reconstructed individual patient data from 37 studies compared neoadjuvant immunochemoradiotherapy versus neoadjuvant chemoradiotherapy in esophageal cancer and found a significant overall survival benefit for the immunotherapy-containing regimen (hazard ratio [HR] 0.71), with pathologic complete response (pCR) of 50% versus 38% for squamous cell carcinoma [15]. The NEO-EC-01 study, a multicenter real-world analysis confirmed favorable safety and survival outcomes for neoadjuvant immunochemoradiotherapy [16]. Both sources derive predominantly from thoracic disease.

The strongest prospective confirmation comes from ESCORT-NEO/NCCES01, the first phase 3 randomized trial of neoadjuvant chemoimmunotherapy in esophageal cancer: in 391 patients with resectable thoracic locally advanced ESCC, neoadjuvant camrelizumab plus albumin-bound paclitaxel and cisplatin raised the pCR rate to 28.0% versus 4.7% with chemotherapy alone (p<0.0001), without increasing postoperative complications [14]. In a parallel comparative-effectiveness study of 1428 thoracic LA-ESCC patients from eight high-volume centers, Guo et al. [17] reported superior 2-year overall survival with neoadjuvant chemoimmunotherapy compared to neoadjuvant chemoradiotherapy (81.3% vs. 71.3%, HR 1.57, p<0.001) after propensity-score matching, despite a lower major pathologic response rate in the chemoimmunotherapy arm (61.5% vs. 71.8%); the survival advantage was driven predominantly by lower distant metastasis (13.5% vs. 25.0%). Both studies exclude cervical disease and should be extrapolated with caution, but together they anchor the indirect evidence base for chemoimmunotherapy as an induction option in cervical ESCC.

Comparative studies suggest neoadjuvant chemoimmunotherapy achieves similar or superior pathologic response compared to neoadjuvant chemoradiotherapy, with potentially lower rates of postoperative complications in selected thoracic series [18,19,20,21,22]. Cervical tumors present distinct anatomic constraints, higher rates of T4 disease at presentation, and greater functional consequences of treatment-related toxicity; applicability therefore remains uncertain. These data derive entirely from thoracic ESCC; given anatomic, functional, and treatment-response differences at the cervical subsite, extrapolation should be treated as hypothesis-generating only.

### 3.3. Chemoimmunotherapy Versus Chemoradiotherapy: Cervical-Specific Considerations

For cervical esophageal cancer, the choice between chemoimmunotherapy and chemoradiotherapy as induction therapy remains unsettled. Chemoradiotherapy has been the established standard for decades, with well-characterized toxicity profiles and established salvage pathways [2,23,24]. Standard preoperative chemoradiotherapy regimens include paclitaxel/carboplatin per the Chemoradiotherapy for Oesophageal Cancer Followed by Surgery (CROSS) protocol and fluoropyrimidine/oxaliplatin-based schedules [8]. Chemoimmunotherapy may deepen pathologic response and avoid some radiation-associated fibrosis, but cervical-specific data remain limited to small series and subgroup analyses [13,25].

Dai et al. [26] recently reported a response-adapted paradigm in 40 cervical esophageal carcinoma patients: two to four cycles of chemotherapy plus a PD-1 inhibitor were delivered as induction, after which patients achieving clinical complete or near-complete response (35.0%; 14/40) proceeded to definitive chemoradiotherapy, while non-responders underwent timely surgery. One-year cancer-specific survival in non-responders favored the surgical arm (93.3% vs. 71.4% for definitive chemoradiotherapy [dCRT], p=0.027) in Dai et al. [26], and the incidence of post-dCRT esophageal fistula differed substantially between responders and non-responders (10% vs. 75%), underscoring the cost of forcing chemoradiotherapy on non-responding disease. Overall laryngeal preservation reached 85% with functional preservation in 77.5%. This is an early cervical-specific attempt to pair chemoimmunotherapy induction with a response-gated decision between dCRT and surgery. It also converges methodologically with the SCENIC trial design [13].

**Figure 2:**
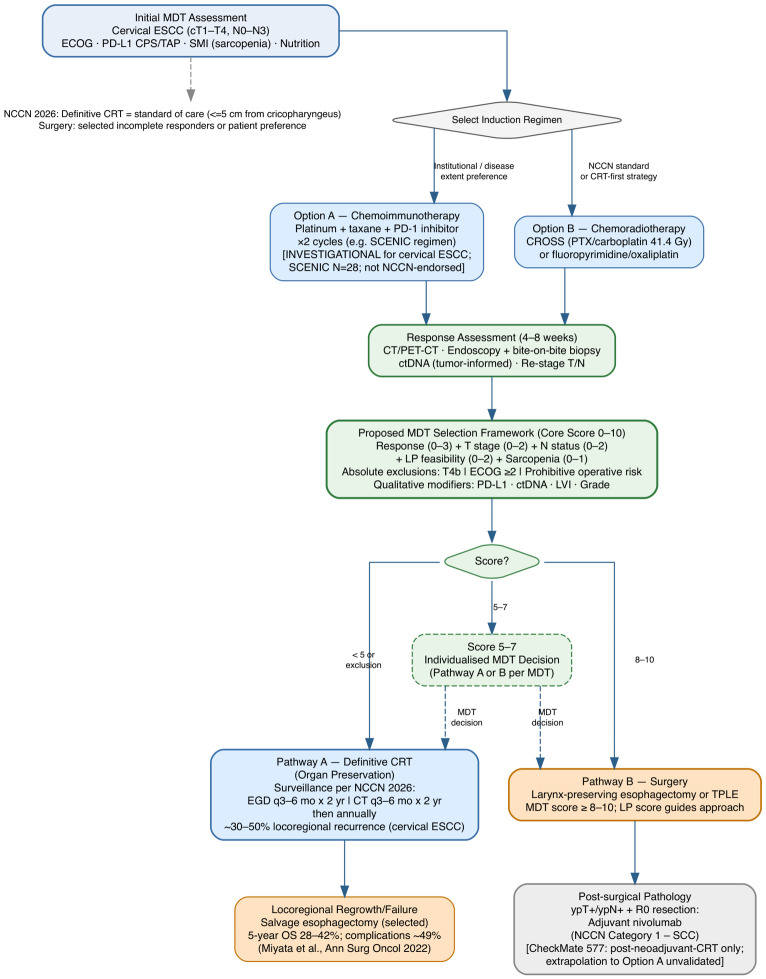
Proposed clinical decision algorithm for cervical esophageal squamous cell carcinoma, integrating induction strategy, response assessment, surgical selection, and postoperative management after R0 resection with residual pathologic disease. Definitive chemoradiotherapy remains the standard pathway; surgery is reserved for selected incomplete responders with resectable disease and acceptable functional feasibility. Adjuvant nivolumab is guideline-supported after preoperative chemoradiotherapy; after PD-1-based induction, adjuvant checkpoint inhibition remains investigational. Solid arrows indicate the primary clinical pathway; dashed arrows indicate conditional or response-dependent transitions. Blue boxes denote induction/treatment steps; green boxes denote response assessment and decision points; orange boxes denote surgical management; grey boxes denote adjuvant/follow-up.

In practice, induction therapy selection depends on disease extent, institutional experience, and the intended balance between response depth and organ preservation. Both pathways are represented in the clinical decision algorithm (Figure 2). The optimal regimen likely turns on baseline resectability, proximity to critical structures, and patient-specific factors including performance status and comorbidities.

## 4. Clinical Outcomes: Surgery After Induction Therapy

### 4.1. Cervical-Specific Survival Data

Cervical-specific survival data are accumulating from multi-institutional databases, single-center series, and systematic reviews [27]. An SEER-based propensity-matched analysis found no significant survival difference between surgery and definitive chemoradiotherapy in 440 cervical esophageal cancer patients for overall survival (OS; HR 0.75, 95% confidence interval [CI] 0.55–1.02, p=0.06), though the study was limited by registry data constraints and lack of response assessment [28]. Patel et al. [2] reported superior 5-year overall survival with definitive therapy (34%) compared with no definitive treatment (13%) in a national database analysis of proximal esophageal squamous cell carcinoma; this comparison is between any definitive treatment and no treatment, not specifically between surgery and chemoradiotherapy. The survival advantage was most pronounced in T3–4 disease. Sabbagh et al. [29] compared neoadjuvant chemoradiotherapy followed by surgery (median OS 31.8 months) with chemoradiotherapy alone (median OS 21.3 months, adjusted HR 0.77, p=0.01) in upper esophageal cancer; this is the more directly relevant comparison for the surgical pathway described in this review. A subsequent full publication with propensity-score matching (N = 386) confirmed an OS benefit for neoadjuvant CRT followed by surgery (mOS 33.2 vs. 20.5 months, HR 1.38, p=0.02), but found no significant survival benefit for cervical tumors (C15.0) specifically; the advantage was driven by thoracic disease [30]. In a cervical-specific single-center series (N = 123), surgery was associated with significantly better progression-free survival (PFS; HR 0.50, p=0.013) and overall survival (HR 0.28, p=0.005) versus chemoradiotherapy for ≥T3 lesions, with induction chemotherapy followed by larynx-preserving surgery achieving the best outcomes [25]. A population-based SEER analysis of 1371 resectable cervical esophageal cancer patients further showed that surgery-based multimodal therapy improved 10-year overall survival versus non-surgical management in both stage strata that were large enough for matched comparison: localized T1–T2N0 disease (20.7% vs. 11.4%, p=0.023) and regional T3–T4 or node-positive disease (20.4% vs. 9.0%, p=0.031) after propensity-score matching [31]. A second SEER cohort by Qi et al. [32] (N = 1329, post-PSM) estimated a smaller and non-significant overall effect for surgery (HR 0.767, p=0.198), illustrating that the apparent benefit in Xu et al. [31] is stage-dependent and does not generalize to an unstratified cervical population. Lu et al. [3] reported complementary evidence from a 270-patient cervical-specific SEER cohort: triple-modality therapy (surgery with chemotherapy and radiotherapy) was associated with substantially lower mortality than single-modality treatment (HR 0.41), reinforcing that any survival signal favoring resection in this disease emerges predominantly within an integrated, multi-modal pathway rather than from surgery used in isolation.

Valmasoni et al. [23] reported a cohort of 97 cervical esophageal cancer patients, finding median overall survival of 42 months with surgery versus 18 months with chemoradiotherapy in non-complete responders, though selection bias likely favored the surgical cohort. Takebayashi et al. [24] found no significant difference in 5-year overall survival between curative surgery (38%) and definitive chemoradiotherapy (32%, p=0.45), but surgery was associated with better local control.

Table 1 summarizes key clinical studies on surgery following induction therapy for cervical esophageal cancer.

**Table 1:** Key studies. Sections A, B, and E list the primary-included cervical-direct studies (n=20) that form the synthesis. Sections C and D list thoracic and meta-analytic sources cited for indirect comparison and biological rationale; these are supporting evidence and are not counted in the included set.

Study, Y	Design	N	Cervical Pts	Regimen	Sx	R0 (%)	pCR (%)	Outcomes	Finding
A. Cervical esophageal cancer: survival comparisons									
Miyakoshi et al., 2022 [25]	Single-center retrospective	123	123	Induction chemotherapy (subset)	Surgery (53) vs. CRT (70)	NR	NR	≥T3: surgery PFS HR 0.50 (p=0.013); OS HR 0.28 (p=0.005)	Surgery associated with better outcomes for ≥T3 disease; best outcomes after induction chemotherapy plus LP surgery
Patel et al., 2023 [2]	NCDB retrospective	2159	2159	Variable (CRT predominant)	Variable	NR	33.3 ^a^	5-year OS: definitive 34% vs. non-definitive 13.3% (p<0.001); surgery HR 0.84 (p=0.08, NS)	Definitive therapy outperformed no treatment; no clear surgery-over-CRT advantage ^a^
Sabbagh et al., 2023 [29]	NCDB retrospective	2310	Cervical + upper third	Neoadj. CRT	Esophagectomy	NR	NR	mOS 31.8 vs. 21.3 mo (p<0.001); adjusted HR 0.77 (p=0.01) ^b^	Trimodality therapy associated with better outcomes overall; no significant T2/T3 subgroup association
Alhalabi et al., 2025 [30]	NCDB retrospective, PSM	386	Mixed (cervical + thoracic)	Neoadj. CRT	Esophagectomy	NR	NR	After PSM: mOS Neoadj. CRT plus surgery 33.2 mo vs. definitive CRT 20.5 mo (p=0.007); HR 1.38 (p=0.02)	Benefit driven by thoracic tumors; no cervical-specific OS benefit
Valmasoni et al., 2018 [23]	Retrospective, 3-arm	148	148	Variable (CRT, chemo)	Surgery alone (56), CRT (52), CRT + surgery (40)	71.9	NR	Non-CR patients: surgery 42 vs. CRT 18 mo (p=0.023); no benefit in CR patients	Surgery associated with better outcomes in non-CR patients; CRT remained appropriate for CR patients
Takebayashi et al., 2017 [24]	Retrospective comparative	49	49	None (primary treatment comparison)	Cervical esophagectomy ± laryngectomy	NR	N/A	5-year OS: surgery 60.6% vs. CRT 51.4% (p=0.89, NS); CRT CR rate 58.3%	OS was comparable; salvage surgery was used for CRT non-responders
Qi et al., 2020 [28]	SEER, propensity-score matched	440	440	NR	Radical surgery	NR	NR	After PSM: HR 0.75 (95% CI 0.55–1.02, p=0.06)	No significant OS difference after matching
Lu et al., 2021 [3]	SEER cohort	347	347	NR	NR	NR	NR	mOS: 14.0 mo (overall)	Registry benchmark showing poor overall prognosis
Xu et al., 2022 [31]	SEER, propensity-score matched	1371	1371	Variable	Surgery-based multimodal	NR	NR	Regional disease: 10-year OS Sx-multimodal 20.4% vs. CRT 9.0% (p=0.031)	Localized disease associated with better outcomes after Sx; regional disease associated with better outcomes after Sx-based multimodal therapy
Qi et al., 2023 [32]	SEER retrospective, 1:1 PSM	440	440	NR (registry)	Radical surgery (84) vs. CRT (356)	NR	NR	Pre-PSM CSS HR 0.955 (0.704–1.295, p=0.766); post-PSM HR 0.767 (0.512–1.149, p=0.198); subgroup T1–T4 all p>0.05	No CSS advantage for radical surgery over CRT after matching; complementary to Xu 2022 which evaluated multimodal surgery
Moon et al., 2022 [33] ^d^	Single-center retrospective (conference abstract)	150	150	Variable	dCRT (100) vs. other incl. surgery (50)	NR	NR	Compares surgery vs. dose-escalated dCRT in resectable CEC 2001–2020; numeric outcomes not yet peer-reviewed	Conference abstract only; supports feasibility of surgery as alternative to dose-escalated dCRT
B. Cervical esophageal cancer: larynx preservation and surgical outcomes									
Yuan et al., 2025 [7]	Retrospective cohort	152	152	Variable	LP (85) vs. TPLE (67)	LP 75.3; TPLE 89.6	NR	2-year OS: LP 71.2% vs. TPLE 43.5% (p=0.035); 90-day mortality: LP 0% vs. TPLE 7.5% (p=0.01)	LP surgery improved OS despite lower R0
Makino et al., 2016 [4]	Retrospective series	100	100	Neoadj. CRT/ chemo (subset)	LP esophagectomy	NR	NR	LP rate: T1–2 90.5%, T3–4 54.3% (responders); LP surgery did not worsen prognosis vs. non-preserving surgery	cT stage and treatment response predicted LP feasibility
Dai et al., 2020 [34]	Retrospective series	15	15	Neoadj. CRT (48 Gy)	LP surgery	53.3	20.0	2-year OS: 50.6%; LP rate: 93.3% (14/15) ^c^	High LP feasibility, but low R0
De Virgilio et al., 2023 [27]	Systematic review, meta-analysis	868	868	Variable	Larynx-preserving (213) vs. non-preserving (229)	NR	NR	Pooled 5-year OS: larynx-preserving 35.1% vs. non-preserving 14.6%; overall 26.6%	LP surgery appeared feasible
Ott et al., 2009 [35]	Single-center retrospective, 1986–2006	109	109	Preop. CRT 94 (86%); primary resection 15 (14%)	Limited resection + free jejunal graft interposition	72.5	26.6	mOS 34.3 mo; 1-/3-/5-year OS 83.8%/47.0%/47.0%; 30-day mortality 1.8%; complete/partial LP 85.3%; reoperation 29.4%	Limited resection with jejunal reconstruction feasible after preop. CRT at high-volume centers; high morbidity offset by oncologic benefit
Katsurahara et al., 2020 [36]	Single-center retrospective, 2000–2016	69	69	Variable (CRT, NAT + Sx, definitive)	LP feasibility by PET-CT cricoid–tumour distance	NR	NR	Cut-off −5 mm: 3-year OS short group 45.4% vs. long group 79.6% (p=0.009); multivariate HR 2.65 (p=0.039)	PET-CT cricoid-distance is an independent prognostic + LP-prediction imaging biomarker
Daiko et al., 2011 [37]	Single-center pilot, 2005–2008	11	11	Post-op. RT 66 Gy + low-dose cisplatin	Cervical esophagectomy ± laryngectomy (adjuvant CRT)	NR	N/A	Grade 3 toxicity: leukopenia 36%, mucositis 9%; 1-/3-year OS 91%/71% (median f/u 39.5 mo)	Adjuvant CRT with low-dose cisplatin tolerable for high-risk (pM1-lym) cervical ESCC; may improve locoregional control
C. Supporting evidence—thoracic ESCC, neoadjuvant chemoimmunotherapy (not counted as included; cited for indirect comparison)									
Zhang et al., 2025 [9]	Prospective cohort, PSM	202	Thoracic	Neoadj. ChemoIO vs. upfront surgery	Esophagectomy	NR	NR	PFS: HR 0.31 (p<0.001); OS: HR 0.42 (p=0.009)	Neoadj. ChemoIO improved PFS and OS vs. upfront Sx
Shao et al., 2025 [19]	Retrospective comparative	397	Thoracic	Neoadj. ChemoIO vs. CRT	Esophagectomy	NR	NR	3-year OS: 73.8% vs. 54.8% (p<0.01)	3-year OS favored ChemoIO over CRT
Chen et al., 2025 [20]	Retrospective 3-way	180	Thoracic	ChemoIO vs. CRT vs. chemo	Esophagectomy	NR	NR	2-year OS: 93.4% vs. 85.2% vs. 78.9%	2-year OS favored ChemoIO
Yan et al., 2026 [16]	Multicenter real-world	89	Thoracic	CRT + immunotherapy	Esophagectomy	NR	50.7	1-year OS: 91.6%; MPR 77.5%	High pCR and MPR with favorable short-term outcomes
Yu et al., 2024 [21]	Retrospective comparative	202	Thoracic	Neoadj. ChemoIO vs. Neoadj. CRT	Esophagectomy	85.2 vs. 92.3	27.5 vs. 36.4	3-year OS: 91.7% vs. 79.8% (p<0.05)	Lower pCR but better OS with ChemoIO than CRT
Wang et al., 2025 [38]	Single-arm	99	Thoracic	ChemoIO	Esophagectomy	99.0	NR	1-year OS: 91.6%; TRG 0–1: 64.7%; grade ≥3 AE 10.1%	Promising single-arm feasibility with very high R0 (99%)
Qin et al., 2024 (ESCORT-NEO) [14]	Multicenter randomized phase 3 randomized controlled trial (RCT)	391	0 (thoracic LA-ESCC)	Cam + nab-TP (132)/Cam + TP (130)/TP (129) × 2 cycles	Esophagectomy + adjuvant Cam	NR	28.0/15.4/4.7	pCR Cam + nab-TP vs. TP Δ23.5% (15.1–32.0, p<0.0001); Cam + TP vs. TP Δ10.9% (p=0.0034); EFS immature; grade ≥3 TRAE 34%/29%/29%	Only phase 3 RCT confirming superior pCR with neoadj. chemoimmunotherapy in LA-ESCC; anchors indirect cervical extrapolation
Guo et al., 2025 [17]	Multicenter prospective-registry, PSM	1428 (PSM 532 + 532)	0 (thoracic LA-ESCC)	NCIT vs. NCRT	Esophagectomy	NR	22.9 vs. 25.9	2-year OS NCIT 81.3% vs. NCRT 71.3% (HR 1.57, p<0.001); 2-year DFS 73.9% vs. 63.4% (HR 1.37, p<0.001); distant mets 13.5% vs. 25.0%; MPR 61.5% vs. 71.8%	Despite lower MPR, NCIT outperforms NCRT in 2-year OS/DFS driven by reduced distant metastasis
D. Supporting evidence—meta-analyses of neoadjuvant chemoimmunotherapy in mixed ESCC (not counted as included; cited for pooled-estimate benchmarks)									
Xu et al., 2023 [10]	Meta-analysis	20 studies	Mixed ESCC	Neoadj. ChemoIO	Esophagectomy	92.8	32.9	Pooled MPR: 58.3%	pCR 32.9%, MPR 58.3%, and R0 92.8% across pooled ESCC studies
Ge et al., 2022 [11]	Meta-analysis	24 trials	Mixed ESCC	Neoadj. ChemoIO	Esophagectomy	NR	31.4	NR	Pooled pCR 31.4%; consistent across studies
He et al., 2023 [12]	Meta-analysis	18 studies	Mixed ESCC	Neoadj. immunotherapy (CRT or chemo)	Esophagectomy	NR	CRT+IO 48%; ChemoIO 29%	NR	Radiotherapy with IO associated with higher pCR
E. Cervical-specific chemoimmunotherapy									
Li et al. (SCENIC), 2024 [13]	Prospective phase II	28	28	Tislelizumab + nab-PTX + carboplatin × 2	Radical esophagectomy (non-responders)	NR	NR	Median follow-up 11 mo; 50% clinical response; grade ≥ 3 AE 16%	First prospective cervical-specific chemoimmunotherapy study
Dai et al., 2024 [26]	Single-center response-adapted cohort	40	40	2–4 cycles chemo + anti-PD-1 induction	Response-adapted: CR/near-CR → dCRT; non-CR → surgery (18) or dCRT (8)	NR	10.0 ^e^	1-year CSS in non-CR: surgery 93.3% vs. dCRT 71.4% (p=0.027); LP 85%, functional LP 77.5%; esophageal fistula 10% (CR-dCRT) vs. 75% (non-CR-dCRT)	Response-adapted paradigm: chemoimmunotherapy induction stratifies to dCRT (LP advantage) or timely surgery (fistula avoidance); first cervical-specific algorithm integrating IO

^a^ pCR rate of 33.3% (40/120) reported in the subgroup of patients who underwent esophagectomy after chemoradiotherapy; the 34% vs. 13.3% OS comparison contrasts any definitive therapy against no definitive treatment, not surgery versus CRT specifically. Surgery did not achieve statistically significant OS superiority over CRT in multivariable or propensity-score analyses (HR 0.84, *p* = 0.08) [2]. ^b^ HR > 1 indicates worse prognosis for definitive chemoradiotherapy relative to neoadjuvant chemoradiotherapy plus surgery [30]. ^c^ N = 15; R0 resection 53.3% (8/15); larynx preservation 93.3% (14/15); survival reported at 2 years (50.6%), not 3 years [34]. ^d^ Moon et al., 2022 [33] is a conference proceedings abstract (IJROBP suppl.ESTRO 2022) with limited numeric reporting; cited for completeness of the evidence scan. Excluded from the weighted summary analysis pending peer-reviewed publication. ^e^ Dai et al. reported clinical complete or near-complete response after induction chemoimmunotherapy in 35.0% (14/40). The pCR value shown in the table refers only to the surgical subset (2/20; 10.0%) and should not be interpreted as the overall cohort response rate [26]. Abbreviations: AE, adverse event; CR, complete response; CRT, chemoradiotherapy; ChemoIO, chemoimmunotherapy; ESCC, esophageal squamous cell carcinoma; HR, hazard ratio; IO, immunotherapy; LP, larynx-preserving; mo, months; mOS, median overall survival; MPR, major pathologic response; N/A, not applicable; NCDB, National Cancer Database; Neoadj, Neoadjuvant; NR, not reported; NS, not significant; OS, overall survival; pCR, pathologic complete response; PFS, progression-free survival; PSM, propensity-score matching; Pts, patients; PTX, paclitaxel; R0, margin-negative resection; SEER, Surveillance, Epidemiology, and End Results; Sx, surgery; TRG, tumor regression grade; TPLE, total pharyngolaryngoesophagectomy.

**Table 2:** Descriptive synthesis of reported outcomes across the cervical-direct primary-included studies. Values are per-study extracted rates, not pooled meta-analytic estimates; denominators differ across outcomes because not every study reported every endpoint. Selection of this subset and the rationale for descriptive rather than quantitative synthesis are described in Methods and the Discussion.

Outcome	*k*	Range (%)	Median (%)	Contributing Studies
R0 resection (surgical cohorts)	7	53.3–98.1	75.3	Dai 2020 [34]; Dai 2024 [26]; Makino 2016 [4] (LP arm); Ott 2009 [35]; Patel 2023 [2] (surgical subgroup); Valmasoni 2018 [23]; Yuan 2025 [7] (LP arm)
Pathologic complete response after induction	4	10.0–33.3	23.3	Dai 2020 [34]; Dai 2024 [26] (surgical subset); Ott 2009 [35]; Patel 2023 [2] (CRT → surgery subset)
Larynx preservation rate (LP-intent cohorts) ^a^	5	43.8–93.3	85.0	Dai 2020 [34]; Dai 2024 [26]; Katsurahara 2020 [36] (surgical subset); Makino 2016 [4]; Ott 2009 [35]
Five-year overall survival (cervical cohorts) ^b^	5	17.4–51.2	41.2	Lu 2021 [3]; Makino 2016 [4] (LP arm); Ott 2009 [35]; Patel 2023 [2] (surgical arm); Xu 2022 [31]
Thirty-day postoperative mortality	4	0.0–3.5	1.9	Dai 2024 [26]; Makino 2016 [4]; Ott 2009 [35]; Patel 2023 [2]

^a^ Restricted to cohorts in which larynx preservation was the stated surgical intent or a pre-planned option; total-resection series are not included. ^b^ When studies reported stratified 5-year OS by modality (Makino 2016 [4]) or by treatment arm (Patel 2023 [2]), the larynx-preserving or surgical value is tabulated. Xu 2022 [31] contributes the overall cervical 5-year OS estimate; its modality-stratified findings are summarized separately in the cervical-specific survival section. Lu 2021 [3] contributes the SEER cervical 5-year OS benchmark. Table 2 is intended to support narrative interpretation, not to replace it. Meta-analytic pooling was not performed because study design, induction regimen, surgical intent, and denominator definitions were too heterogeneous to justify a single effect estimate; this limitation is addressed in Methods and the Discussion.

### 4.2. Pathologic Response and Survival Correlation

Pathologic complete response after neoadjuvant therapy is consistently associated with improved survival in esophageal cancer. In cervical-specific series, pathologic complete response rates after chemoradiotherapy range from 25% to 35% [4,34]. The SCENIC trial reported an interim clinical response signal with neoadjuvant chemoimmunotherapy in cervical disease, but pathology-confirmed responses were not reported and long-term survival data are not yet mature [13].

The Surgery As Needed for Oesophageal cancer (SANO) trial established that active surveillance following neoadjuvant chemoradiotherapy was non-inferior to standard esophagectomy with respect to 2-year overall survival (74% vs. 71%) in patients achieving clinical complete response; isolated locoregional regrowth occurred in 48% of the surveillance arm [39]. A subsequent Markov decision analysis found that standard surgery was favored at the 5-year horizon when overall recurrence exceeded 43%, whereas active surveillance was favored only when locoregional recurrence constituted more than 94% of all recurrences [40]. These data are not cervical-specific and are most relevant as context for complete responders, not as direct support for surgical consolidation in cervical disease.

Pathologic response assessment is only possible after surgical resection. The preSANO cohort study demonstrated that bite-on-bite endoscopic biopsies, in combination with clinical and radiologic assessment, improve locoregional response detection; PET/CT is primarily useful for identifying interval distant metastases rather than confirming local complete response [41]. A prospective squamous-specific extension followed in the preSINO trial, supporting the relevance of structured response evaluation in squamous histology while still leaving cervical-specific performance uncertain [42]. Patients with complete clinical response may be managed non-operatively in appropriate settings. Patients with incomplete response present the harder problem: the literature supports multidisciplinary reassessment, not a uniform rule.

### 4.3. Larynx Preservation: Feasibility and Outcomes

Larynx-preserving surgery has expanded the surgical options for cervical esophageal cancer. Makino et al. [4] reported 100 consecutive larynx-preserving esophagectomies, achieving larynx preservation in 90% of T1–2 tumors and 54% of T3–4 tumors. Five-year overall survival was 58% in the larynx-preserved group versus 31% in the total laryngectomy group; this difference likely reflects tumor biology rather than surgical technique. The same series identified an induction-dose signal: disease-free survival after preoperative 60 Gy was 66.7% versus 27.8% with 40 Gy, suggesting that the preoperative prescription is itself a determinant of subsequent outcome when larynx preservation is the planning goal.

Yuan et al. [7] reported 2-year overall survival of 71.2% for larynx-preserving surgery versus 43.5% for total pharyngolaryngoesophagectomy, with higher R0 rates in the pharyngolaryngoesophagectomy group (89.6% vs. 75.3%). The survival difference in that series most plausibly reflects selection bias toward more advanced tumors requiring total resection rather than any inherent oncologic inferiority of laryngectomy. When larynx preservation is not technically feasible, total pharyngolaryngoesophagectomy remains an oncologically valid option and should not be regarded as a failure of surgical selection.

Ott et al. [35] provide an older but methodologically coherent single-center reference point from Heidelberg (N = 109, multimodal preoperative therapy followed by transthoracic resection): R0 resection was achieved in 72.5%, larynx preservation in 85.3%, and median overall survival was 34.3 months, with preoperative radiotherapy at 45 Gy associated with the best survival subset. The series pre-dates contemporary immunotherapy but remains one of the larger single-institution experiences supporting that R0 cervical resection with larynx preservation is reproducibly achievable when anatomy and induction response cooperate.

Preoperative selection for larynx preservation has begun to move from surgical judgement alone to quantitative imaging. Katsurahara et al. [36] showed on pretreatment PET/CT that a tumor-to-cricoid distance below −5 mm independently predicted worse overall survival (HR 2.65, 95% CI 1.04–8.09, p=0.039), with 3-year overall survival of 45.4% in the short-distance group versus 79.6% in the long-distance group (p=0.009). This threshold is used prospectively in our proposed framework (Section 6) as the anchor for the laryngeal preservation feasibility weight.

Dai et al. [34] reported neoadjuvant chemoradiotherapy (48 Gy) followed by larynx-preserving surgery in 15 cervical esophageal cancer patients, achieving pathologic complete response in 20%, larynx preservation in 93.3% (14/15), R0 resection in 53.3% (8/15), and 2-year overall survival of 50.6%. The low R0 rate underscores the importance of case selection and preoperative margin assessment. In appropriately selected patients with favorable response to induction therapy, larynx preservation is feasible and associated with acceptable oncologic outcomes.

Daiko et al. [37] piloted a different question—whether adjuvant chemoradiotherapy after larynx-preserving surgery could recover outcome in patients with M1-lymph-node involvement—in a small cohort of 11 patients. The numerical yield is limited by sample size, but the study establishes that planned postoperative consolidation is feasible after laryngeal preservation and is reported here for completeness rather than as comparative evidence.

### 4.4. Surgical Timing After Induction Therapy

Optimal timing of surgery after neoadjuvant therapy remains debated. For chemoradiotherapy, the conventional interval is 4–8 weeks, balancing maximal tumor response against progressive radiation-induced fibrosis. For chemoimmunotherapy, the optimal interval is less defined. Kita et al. [43] analyzed JCOG1109 data and found no significant difference in surgical or oncologic outcomes between early (4–6 weeks) and delayed (7–12 weeks) surgery after neoadjuvant chemotherapy.

Fang et al. [44] found that surgery within 4–8 weeks after neoadjuvant chemoimmunotherapy was associated with lower postoperative complication rates compared to delayed surgery (>8 weeks), with no difference in pathologic response or survival; similar conclusions were reported in a subsequent analysis by Fang [45]. Available retrospective data do not clearly support departing from conventional 4–8 week surgical timing, though this remains unsettled and may depend on the specific immunotherapy regimen, depth of response, and patient-specific factors.

## 5. Surgical Techniques and Perioperative Considerations

### 5.1. Surgical Approaches

NCCN guidelines specify that esophagectomy for squamous cell carcinoma is indicated for non-cervical esophageal disease; for cervical tumors within 5 cm of the cricopharyngeus, definitive chemoradiotherapy is the preferred approach [8]. When surgery is selected for cervical disease after induction therapy, the goals are R0 resection with larynx preservation where anatomically feasible.

Traditional approaches required total pharyngolaryngoesophagectomy with permanent tracheostomy, but modern techniques emphasize larynx preservation when oncologically feasible [1,4,5,46]. Larynx-preserving techniques include partial pharyngectomy with cervical esophagectomy, preserving the larynx when adequate proximal clearance with microscopically negative margin can be achieved without compromising resection [4,5]. Abe et al. [46] described a hybrid endoscopy-assisted approach in which iodine staining during endoscopy precisely defines the proximal mucosal extent of tumor, facilitating margin identification in cases where the boundary is otherwise difficult to delineate; negative proximal margins were achieved in 5 of 6 patients with no aspiration pneumonia postoperatively. Reconstruction options include gastric pull-up, free jejunal interposition, or colonic interposition; gastric pull-up is most common due to single anastomosis and reliable blood supply [1,5].

Minimally invasive approaches remain technically challenging for cervical tumors given limited working space and proximity to critical neurovascular structures [47]. Hybrid approaches combining open cervical dissection with minimally invasive thoracic mobilization may offer a compromise between oncologic adequacy and reduced morbidity. Emerging platforms combining preoperative 3D anatomical reconstruction with robotic-assisted minimally invasive resection may improve margin assessment and reduce morbidity in anatomically complex cervical tumors [48]; the supporting evidence base derives from thoracic esophagectomy cohorts and applicability to the cervical subsite remains to be established. Where larynx-preserving resection is planned, intraoperative frozen-section assessment of the proximal mucosal margin is reasonable to confirm an R0 plane before reconstruction, particularly when the post-induction tumour bed is fibrotic or anatomically unclear [5,46].

### 5.2. Perioperative Outcomes and Complications

Perioperative outcomes are influenced by tumor location, extent of resection, reconstruction technique, and prior induction therapy. Table 5 summarizes major complications comparing surgery versus definitive chemoradiotherapy.

Anastomotic leak rates range from 6% to 15%, higher than thoracic anastomoses due to compromised blood supply and tension on the cervical anastomosis [38,49]; most leaks are managed conservatively with salvage rates exceeding 90%. Recurrent laryngeal nerve injury occurs in 15–30% of cases [1,50]. Aspiration pneumonia, occurring in 15–35% of patients, relates to recurrent laryngeal nerve injury, anastomotic stricture, and baseline swallowing dysfunction [49,50]. Postoperative vasopressor requirement is infrequently reported in cervical-specific series but is a recognized perioperative risk in this extended resection [1,38]. Given the high rate of pretreatment dysphagia, induced weight loss, and anastomotic complications in this cohort, placement of a feeding jejunostomy at the time of surgery has been used selectively to protect nutritional status during the early postoperative period and to bridge enteral intake in the event of anastomotic leak or delayed oral resumption; the cervical-specific evidence remains limited and the decision should be individualized within the multidisciplinary plan [1,35].

Thirty-day mortality ranges from 3% to 8% in contemporary series, with lower rates at high-volume centers [2,4,7]. Postoperative complication rates after neoadjuvant chemoimmunotherapy appear comparable to or slightly lower than after chemoradiotherapy in selected thoracic esophageal squamous cell carcinoma series, though cervical-specific data remain limited [18,22,51].

## 6. Patient Selection: A Proposed Multidisciplinary Framework

### 6.1. Rationale for Structured Patient Selection

No validated scoring system exists for cervical esophageal cancer. We propose a practical multidisciplinary framework to support structured decision-making, recognizing that it is an author-derived conceptual tool rather than a validated clinical instrument. The framework is designated as a “proposed multidisciplinary team (MDT) selection framework” to avoid implying established validity; prospective validation is required before it can be used as a standardized instrument.

The domain weights in the core score are anchored to a prespecified evidence-to-decision weighting that prioritizes cervical-direct over thoracic-indirect inputs (Supplementary Materials Table S9). Each reflects the prognostic signal carried by that variable in the best cervical-specific comparative data available, and each is anchored where possible to an explicit published threshold rather than to expert intuition alone.

Response to induction therapy receives the highest weight (maximum 3 of 10), but only when residual disease remains biopsy-confirmed and resectable. Miyakoshi et al. [25] found that the survival advantage of surgery was confined to patients with at least a partial response and ≥T3 disease; Valmasoni et al. [23] reported that the benefit of resection was concentrated in non-complete responders, with complete responders managed non-operatively showing no additional gain. Dai et al. [26] extended this logic in a response-adapted cohort: among non-complete responders, planned surgery yielded a 1-year cancer-specific survival of 93.3% versus 71.4% with continued dCRT (p=0.027); complete or near-complete responders were triaged to dCRT, so comparative surgical benefit in that subgroup cannot be inferred. Response therefore behaves less like one prognostic covariate among several and more like a gate that re-opens the surgical question after induction.

Post-induction T and N stage each receive 2 points. The strongest post-matching estimate for this weighting comes from Xu et al.’s cervical-restricted SEER analysis [31]: after propensity-score matching, surgery-based multimodal therapy outperformed non-surgical management in both stage strata that were large enough to analyze separately: 10-year overall survival 20.7% versus 11.4% in localized T1–T2N0 disease (p=0.023) and 20.4% versus 9.0% in regional T3–T4 or node-positive disease (p=0.031). The effect is not uniform across the stage spectrum, which is exactly what a graded weighting scheme should capture; residual disease burden and persistent nodal disease both meaningfully reduce the probability of R0 benefit at this anatomic level, but neither abolishes it while resectability is preserved.

Laryngeal preservation feasibility carries equal weight to T and N staging (2 points) because at the cervical subsite, functional outcome cannot be separated from oncologic planning, and because a reproducible imaging-based threshold now exists. Katsurahara et al. [36] showed on pretreatment PET/CT that a tumor-to- cricoid distance below −5 mm independently predicted worse overall survival (HR 2.65, 95% CI 1.04–8.09, p=0.039; 3-year OS 45.4% for short-distance vs. 79.6% for long-distance tumors, p=0.009), a finding that converts “proximity to the larynx” from a qualitative impression into an operable cutoff. Yuan et al. [7] provide the complementary functional signal: larynx-preserving resection achieved a 2-year overall survival of 71.2% versus 43.5% with total pharyngolaryngoesophagectomy, at a broadly comparable R0 rate. Makino et al. [4] add a dose-related modifier from the induction side: disease-free survival after preoperative 60 Gy was 66.7% versus 27.8% with 40 Gy, supporting the view that larynx preservation is a plannable outcome when the anatomy and the induction prescription are aligned, not an opportunistic one.

Sarcopenia receives the minimum positive weight (1 point). It is a modifiable variable that informs perioperative risk and prehabilitation candidacy; the cervical-specific evidence for sarcopenia as an independent determinant of oncologic benefit does not yet match the magnitude carried by response or stage, and the weight reflects that asymmetry.

These weights are clinically derived, not statistically optimized. They summarize the authors’ reading of the cervical-specific evidence and require prospective calibration before the framework can be applied as a validated instrument. No discrimination, calibration, decision-curve, inter-rater agreement, adherence, or time-to-decision metrics are reported in this review; feasibility and inter-MDT reliability require prospective testing.

### 6.2. Proposed Framework Components

Table 3 summarizes the core clinical, radiologic, and physiologic selection framework following induction therapy. Histopathologic and biologic modifiers, functional requirements, and framework interpretation are presented in Table 4.

**Table 3:** Core multidisciplinary selection score after induction therapy (0–10 points). Absolute exclusion criteria override the score. See footnotes for measurement details.

Criterion	Assessment	Weight
Section A. Clinical, radiologic, and physiologic criteria (core score 0–10)		
Response to induction therapy	Biopsy-confirmed resectable residual disease with major response (>50% reduction)	3
	Biopsy-confirmed resectable residual disease with minor response (<50% reduction)	2
	Complete clinical response with negative bite-on-bite biopsy	Organ-preservation pathway; not scored for surgery
	Stable/progressive disease without R0 feasibility	0
Post-induction T stage	T1–T2	2
	T3	1
	T4a	0
	T4b: unresectable invasion	Exclusion *
Post-induction nodal status	N0	2
	N1	1
	N2–N3	0
Laryngeal preservation feasibility ^†^	Negative proximal margin achievable with laryngeal preservation; tumor-to-cricoid distance ≥−5 mm where measurable	2
	Borderline anatomy, including tumor-to-cricoid distance <−5 mm or close cricoid/laryngeal margin	1
	Laryngeal preservation not feasible	0
Sarcopenia status ^‡^	Non-sarcopenic (skeletal muscle index [SMI] above sex-specific cutoff on staging CT)	1
	Sarcopenic (SMI below cutoff); refer for prehabilitation	0
Absolute exclusion criteria (override any score)		
T stage	T4b disease (unresectable) *	Exclusion
Performance status	Eastern Cooperative Oncology Group (ECOG) performance status ≥ 2	Exclusion
Physiologic reserve ^§^	Prohibitive operative risk on validated assessment (eg, Estimation of Physiologic Ability and Surgical Stress Comprehensive Risk Score [E-PASS CRS] above institutional cutoff)	Exclusion

* T4b is listed under both the T-stage scoring domain (score 0, exclusion flag) and the absolute exclusion section for emphasis; the exclusion overrides any calculated score. ^†^ A score of 0 for laryngeal preservation feasibility indicates that total pharyngolaryngoesophagectomy would be required. This does not exclude surgery, but it carries greater functional morbidity and should be weighed explicitly in multidisciplinary discussion; Yuan et al. [7] reported R0 resection of 89.6% and 2-year overall survival of 43.5%. ^‡^ Sarcopenia is assessed by skeletal muscle index at L3 on staging CT using sex-specific cutoffs [52,53]. A score of 0 does not exclude surgery but should prompt prehabilitation and explicit MDT review. When L3 is unavailable, T12-level SMI may be used as a surrogate [54]. ^§^ Physiologic reserve should be assessed with a validated tool. E-PASS CRS, the Esophageal Vitality Index, or geriatric assessment may be used according to institutional practice [55,56,57]. ECOG ≥ 2 is treated here as a binary exclusion threshold. Section A core score range: 0–10. The five scored domains yield: Response (max 3) + T stage (max 2) + Nodal status (max 2) + LP feasibility (max 2) + Sarcopenia (max 1) = 10.

**Table 4:** Qualitative modifiers, mandatory functional criteria, and interpretation of the proposed framework.

Criterion	Assessment	Interpretation
Section B. Histopathologic, endoscopic, and biologic modifiers (qualitative)		
Endoscopic-biopsy response ^a^	Near-complete response on endoscopy/biopsy	Favorable
	Residual disease on bite-on-bite biopsy still amenable to R0 resection	Favorable
	Poor response or uncertain resectability on endoscopy	Unfavorable
Tumor differentiation grade ^b^	Well or moderately differentiated on pretreatment biopsy	Favorable
	Poorly differentiated or high Cellular Dissociation Grade	Unfavorable
Lymphovascular invasion (LVI) ^c^	Absent on endoscopic resection specimen (if available)	Favorable
	Present, or suspected from high-risk endoscopic features	Unfavorable
PD-L1 expression (baseline) ^d^	Combined positive score (CPS) ≥ 10 or tumor area positivity (TAP) ≥ 10%	Favorable
	CPS < 10/TAP < 10%	Neutral
circulating tumor DNA (ctDNA) clearance (if available) ^e^	Undetectable on tumor-informed testing 4–6 wk after induction	Favorable
	Persistent post-induction detectability	Unfavorable
Section C. Functional criteria (mandatory requirements)		
Physiologic reserve	Adequate for planned resection on validated assessment	Required
	Prohibitive operative risk	Exclusion
Nutritional status	Albumin ≥ 3.5 g/dL and weight loss < 10%	Required
	Albumin < 3.5 g/dL or weight loss > 10%	Requires optimization
Swallowing function	Oral intake preserved without major aspiration	Favorable
	Enteral feeding dependence or recurrent aspiration	Requires evaluation
Framework interpretation		
8–10 points	MDT review may favor surgery	Core score
5–7 points	Individualized MDT decision	Core score
<5 points	Favor definitive CRT or organ-preserving strategies	Core score
Absolute contraindications	T4b disease, ECOG ≥ 2, or prohibitive operative risk on validated assessment	Override

^a^ Endoscopic bite-on-bite biopsies are recommended for locoregional residual disease detection; PET/CT is used mainly to exclude interval distant metastases [41]. Definitions of incomplete response varied across included cohorts, and a cervical-specific diagnostic accuracy standard has not been established. ^b^ Tumor differentiation grade should be assessed on the pretreatment biopsy. Cellular Dissociation Grade may add prognostic information beyond routine WHO grading [58,59]. ^c^ LVI is an independent prognostic factor after neoadjuvant therapy but is reliable only on resection or endoscopic resection specimens; biopsy surrogates are indirect [60,61,62]. ^d^ PD-L1 should be assessed with a prespecified assay and scoring system, preferably 22C3 CPS or SP263 TAP where available. High PD-L1 expression (CPS ≥ 10 or TAP ≥ 10%) is associated with better response to immunotherapy. CPS and TAP show high concordance, but cervical-specific thresholds are not validated; this remains a qualitative modifier, not a stand-alone decision rule [8,63,64]. ^e^ Tumor-informed panels are preferred over tumor-agnostic approaches; sampling is recommended 4–6 weeks after induction completion. Negativity should be defined a priori as no tracked variants above the laboratory limit of detection. Persistent detectability is unfavorable, but cervical-specific decision thresholds are not validated; ctDNA does not alter the core score and should not be used in isolation [65,66,67,68,69]. Sections B and C modify, but do not numerically change, the Section A core score. This framework is author-derived and requires prospective validation; biomarker modifiers remain exploratory until cervical- specific assays, thresholds, and net-benefit estimates are established. Abbreviations: CPS, combined positive score; CRS, Comprehensive Risk Score; ctDNA, circulating tumor DNA; ECOG, Eastern Cooperative Oncology Group; LVI, lymphovascular invasion; PD-L1, programmed death-ligand 1; TAP, tumor area positivity.

### 6.3. Application in Clinical Practice

The proposed framework is intended to support multidisciplinary decision-making rather than replace clinical judgment. Patients with core scores of 8–10 may be considered for surgery, particularly when larynx preservation is feasible and endoscopic biopsy confirms residual disease amenable to complete resection. Scores of 5–7 warrant individualized evaluation, balancing potential oncologic benefit against functional morbidity and patient preferences. Scores below 5, or the presence of absolute contraindications, generally favor definitive chemoradiotherapy or non-surgical management.

Sarcopenic patients identified on staging CT (SMI below sex-specific cutoff) should be referred for prehabilitation assessment before surgical decision-making; structured exercise and nutritional intervention are potentially modifiable interventions that may improve perioperative outcomes [52,53].

Surgical outcomes are volume-dependent; this framework assumes treatment at an experienced high-volume center (typically ≥20 esophagectomies per year). At lower-volume centers, threshold scores for surgical candidacy may require upward adjustment, and referral to a specialized center should be considered [55].

#### 6.3.1. Illustrative Example

A 62-year-old patient with cT3N1 cervical esophageal squamous cell carcinoma (ECOG 0, PD-L1 CPS 15, non-sarcopenic) receives two cycles of platinum/taxane plus PD-1 inhibitor. Post-induction CT and endoscopy demonstrate residual disease on biopsy, partial response exceeding 50%, downstaging to ypT2N0, with laryngeal preservation anatomically feasible. Core framework score: response 3 + T stage 2 + nodal status 2 + LP feasibility 2 + sarcopenia 1 = 10 points. No absolute exclusions are met. Section B modifiers: favorable PD-L1 expression (CPS 15), favorable endoscopic biopsy (residual disease confirmed amenable to resection). The framework interpretation supports surgical evaluation after multidisciplinary review; the high CPS does not redirect toward organ preservation because residual disease is present on biopsy.

If post-induction ctDNA were persistently detectable on a tumor-informed panel, this would represent an unfavorable biologic modifier requiring explicit multidisciplinary discussion but would not automatically exclude surgery in a patient with a 9-point core score and resectable disease.

#### 6.3.2. Post-Surgical Management

Patients undergoing surgery after preoperative chemoradiotherapy who have residual pathologic disease (ypT+ and/or ypN+ with R0 resection) should be considered for adjuvant nivolumab (NCCN category 1 recommendation for squamous cell carcinoma) [8]. This step is absent from most published algorithms and institutional frameworks but represents current standard of care; it should be incorporated into the post-surgical management pathway. After PD-1-based induction, however, the incremental benefit of additional adjuvant checkpoint inhibition is not established in cervical ESCC. Thoracic data such as ESCORT-NEO include adjuvant PD-1 after PD-1-based induction, but survival outcomes remain immature and cervical applicability is uncertain. Re-exposure should therefore be considered investigational and discussed in a trial-oriented MDT setting rather than applied automatically.

Pathologic complete response cannot be determined preoperatively. Endoscopic-biopsy response therefore represents the most informative available surrogate for treatment response and plays a central role in assessing surgical candidacy.

## 7. Clinical Decision Algorithm

Figure 2 presents a proposed clinical decision algorithm integrating initial staging, induction therapy selection, response assessment, and treatment allocation for conceptual guidance. Feasibility, adherence, time-to-decision, and inter-MDT reliability were not evaluated. This algorithm reflects current practice patterns but acknowledges substantial institutional variation and evolving evidence. The algorithm depicts two parallel induction options: chemoimmunotherapy (platinum, taxane, PD-1 inhibitor) and chemoradiotherapy (per CROSS or fluoropyrimidine-based protocols), reflecting the reality that neither pathway is established as superior for cervical disease and that induction selection depends on institutional practice, disease extent, and whether organ-preservation intent guides initial therapy planning.

## 8. Comparative Effectiveness: Surgery Versus Definitive Chemoradiotherapy

### 8.1. Survival Outcomes

**Figure 3:**
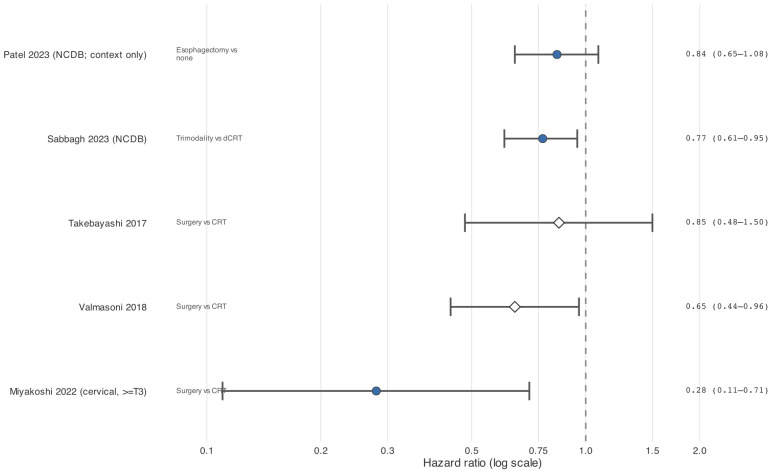
Study-level hazard ratios for overall survival in comparative studies of surgery and definitive chemoradiotherapy in cervical esophageal cancer. Filled circles denote directly reported estimates; open diamonds denote estimates approximated from published Kaplan–Meier curves. This figure is a descriptive synthesis, not a meta-analysis. Studies shown: Miyakoshi 2022 [25]; Valmasoni 2018 [23]; Takebayashi 2017 [24]; Sabbagh 2023 [29]; Patel 2023 [2].

Figure 3 presents a descriptive visual synthesis of reported study-level effect estimates from cervical-specific comparative studies; the underlying study-level source data are tabulated in Supplementary Materials Table S10. Hazard ratios and confidence intervals were extracted from published reports or approximated from published Kaplan–Meier curves where numerical estimates were not directly reported. The figure emphasizes cervical-relevant comparative evidence, including the cervical-specific Miyakoshi series and registry-based analyses from Sabbagh, Patel, Valmasoni, and Takebayashi [2,23,24,25,29]. Because the studies differ substantially in design, comparison groups, and underlying populations, the figure is intended as a descriptive synthesis rather than a poolable meta-analytic dataset.

The available data suggest an association between surgery and improved survival in selected patients; however, heterogeneity, selection effects, and unaddressed time-related biases preclude causal inference [70]. Patients selected for surgery have better performance status, more favorable tumor biology, and access to experienced surgical teams; the survival difference may reflect those characteristics as much as the treatment itself. Time-origin definitions also vary across studies, and the descriptive synthesis did not model surgery as a time-dependent exposure. Patients must survive and remain fit long enough to undergo resection, so immortal-time bias may overestimate surgical benefit, particularly when estimates are approximated from Kaplan–Meier curves.

Within the definitive chemoradiotherapy arm itself, dose intensity is an under-acknowledged source of comparative heterogeneity. Moon et al. [33] reported in conference form that patients receiving ≥59.4 Gy achieved a 3-year overall survival of 65.4% versus 51.7% with lower-dose dCRT (p=0.046); because the report is an abstract and the full peer-reviewed analysis is pending, the estimate is treated as provisional, but it is consistent with the Makino dose-finding signal and argues against interpreting all “dCRT” comparators as a single uniform arm.

### 8.2. Functional Outcomes and Quality of Life

**Figure 4:**
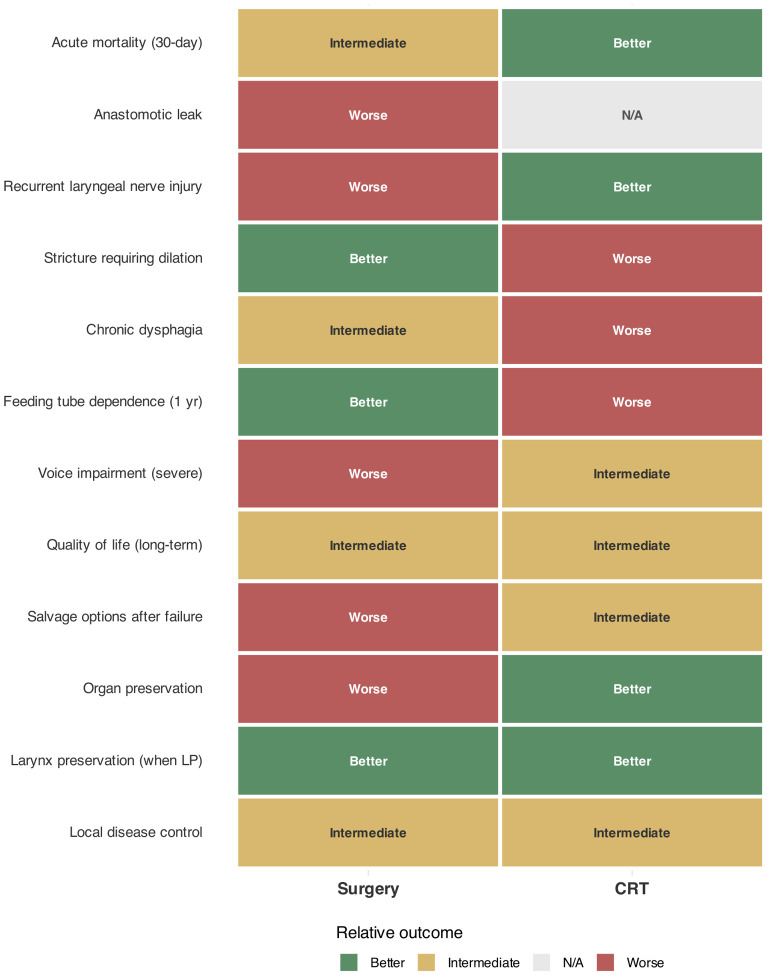
Qualitative heatmap comparing functional outcomes of surgery and definitive chemoradiotherapy in cervical esophageal cancer across peri-treatment morbidity, long-term swallowing outcomes, organ preservation, and salvage feasibility. Shading is qualitative and intended for comparative visualization rather than quantitative inference.

Figure 4 presents a heatmap of functional outcomes comparing surgery versus chemoradiotherapy across multiple domains; the evidence basis underpinning the qualitative shading is documented in Supplementary Materials Table S11. Surgery carries higher acute morbidity (recurrent laryngeal nerve injury, anastomotic leak), but is associated with better long-term swallowing function and lower rates of chronic stricture in surviving patients. Chemoradiotherapy offers organ preservation but is associated with significant rates of chronic dysphagia and pharyngo-oesophageal stricture (37–52% after CRT; 76% after RT alone in a 20-year series) [71], higher feeding tube dependence, and limited salvage options for local recurrence. Stricture risk is highest at the cervical/hypopharyngeal subsite, driven by post-cricoid mucosal apposition and proximity to the pharyngeal constrictors; taxane-based CRT carries higher stricture rates than platinum-based regimens [72].

Quality-of-life data in cervical esophageal cancer are sparse. In thoracic disease, the CROSS trial showed that neoadjuvant chemoradiotherapy did not adversely affect postoperative health-related quality of life compared with surgery alone, with impairment in physical functioning and fatigue persisting at one year in both groups; the long-term CROSS data showed no differential effect at median 105-month follow-up [73,74]. Whether these patterns apply to cervical disease is uncertain. Surgery after induction therapy for cervical cancer is associated with acute functional decline from anastomotic complications, recurrent laryngeal nerve injury, and swallowing dysfunction; recovery trajectories are poorly characterized in published series.

Perioperative complications relevant to recovery and chemotherapy tolerance are summarized in Table 5. Surgery-specific risks include anastomotic leak, recurrent laryngeal nerve injury, aspiration pneumonia, and vasopressor-requiring hemodynamic instability. Each of these affects the patient’s capacity to receive any planned adjuvant or salvage treatment and should be factored into preoperative risk-benefit discussions.

**Table 5:** Major complications of surgery versus definitive chemoradiotherapy in cervical esophageal cancer.

Complication	Surgery (%)	CRT (%)	*p*-Value	Notes
Anastomotic leak	6.1–15.0	N/A	–	Higher with cervical anastomosis; salvage usually feasible [38,49]
Pulmonary or aspiration events	10–35	8–25	NS	Overlapping respiratory and swallowing risk in both groups
Recurrent laryngeal nerve injury/severe voice impairment	15.4–30.0	5–20	<0.05	Often transient but functionally important [1,50]
Stricture requiring dilation	8–20	37–52	<0.01	CRT-associated strictures more common; cervical/hypopharyngeal subsite at highest risk [71,72]
Tracheoesophageal fistula	7.7 (perioperative)	5–15 (late)	NS	Surgery: early and manageable; CRT: late and often severe
30-day mortality	3–8	1–3	NS	Volume-dependent; high-volume centers < 5% [2,4,7]
Grade ≥ 3 adverse events	10.1–27.8	15–30	NS	Immunotherapy arm: 16–27.8%; chemotherapy alone: 10–20%
Long-term swallowing/ feeding-tube burden	10–20	25–40	<0.01	Persistent dysphagia is more common after CRT
Quality of life (EORTC QLQ-C30)	Acute decline with partial recovery	Long-term impairment data limited for cervical disease	–	Cervical-specific longitudinal QoL data are sparse; thoracic CROSS patterns may not generalize [73,74]
Salvage treatment feasibility	CRT salvage: 40–60% eligible	Surgery salvage: 10–20%	<0.01	Primary surgery preserves CRT salvage option; salvage esophagectomy after failed primary CRT achieves 5-year OS 28–42% in selected series, with complication rate ∼49% [75]

Estimated vasopressor requirement after surgery (10–20%) is reported inconsistently in cervical-specific series and is therefore not shown as a separate row. CRT, chemoradiotherapy; EORTC QLQ-C30, European Organisation for Research and Treatment of Cancer Quality of Life Questionnaire Core 30; N/A, not applicable; NS, not significant (p>0.05); RLN, recurrent laryngeal nerve. Surgery data derived from Liu et al. [50] (TEF 7.7%, RLN 15.4%, aspiration 23.1%); Wang et al. [38] (leak 6.1%, grade ≥ 3 10.1%); Li et al. [13] (grade ≥ 3 16%). CRT stricture rate range (37–52%) from Hamer et al. [71] (52% after CRT, 76% after RT alone; 32% required dilation) and Prisman et al. [72] (37% symptomatic stricture overall; cervical/hypopharyngeal subsite highest risk). Salvage esophagectomy outcomes from Miyata et al. [75] (227 patients; 5-year OS 28% persistent vs. 42% recurrent disease). CRT data extrapolated from Chichevatov et al. [76] and comparative studies [23,24,28].

## 9. Discussion

Cervical esophageal cancer still sits in an awkward space between guideline default and surgical judgment. Definitive chemoradiotherapy remains the standard starting point. That is not in dispute. The real question comes later, after induction, when residual disease persists but remains technically resectable. The literature does not settle that question cleanly. It does, however, show a recurring pattern. Surgery appears to matter most in incomplete responders selected carefully for resection at experienced centers. The signal is not uniform. It is also not easy to dismiss.

The cervical-specific datasets are small and mostly retrospective, but they do not all cancel each other out. Miyakoshi et al. [25] reported better progression-free and overall survival for surgery in ≥T3 disease after induction chemotherapy. Registry studies and cohort series suggest a similar possibility [2,23,24,31]. That said, the usual problem remains intact. Patients selected for surgery are not exchangeable with those treated non-operatively. They tend to have better functional reserve, more favorable anatomy, and access to teams that can deliver complex resection with acceptable morbidity. The observed advantage may reflect treatment effect, selection, or both.

This review does not identify a single new prognostic factor. Response depth, post-induction stage, frailty, PD-L1 expression, and ctDNA dynamics are already familiar concepts in esophageal oncology, and most biomarker evidence still comes from thoracic ESCC. The contribution is their integration into a cervical-specific MDT selection framework that makes the tradeoffs explicit: oncologic response and post-induction stage are considered alongside laryngeal preservation feasibility, sarcopenia, and exploratory molecular modifiers. This matters because cervical disease is anatomically different from thoracic disease. The decision is not simply whether a tumor is resectable, but whether resection can deliver meaningful local control without unacceptable loss of voice, swallowing function, or physiologic reserve. In that sense, the framework is best understood as a structured research and MDT communication tool rather than a validated calculator. The framework has not been prospectively validated, and the domain weights are anchored to retrospective and predominantly thoracic-extrapolated data; it is therefore presented as a hypothesis-generating, conceptual aid for multidisciplinary discussion and not as a clinically validated selection instrument.

Margin status likely sits near the center of this issue. Data from thoracic squamous cell carcinoma support the importance of local control and R0 resection after induction treatment [77,78], but cervical anatomy makes that endpoint harder to achieve and more costly in functional terms. Proximity to the larynx, trachea, and great vessels narrows the range of technically meaningful surgery. That is why laryngeal preservation feasibility and residual resectability carry so much weight in the proposed framework. They are not minor technical details. They are often the decision.

The surveillance literature is useful, but only up to a point. SANO showed non-inferiority of active surveillance for 2-year overall survival in complete clinical responders, with better short-term health-related quality of life in the surveillance arm [39,79]. None of that resolves the cervical setting. Salvage anatomy is less forgiving here. Local regrowth does not carry the same practical meaning at the thoracic and cervical levels. The Bondzi-Simpson re-analysis [40] underscores how sensitive the long-term balance is to recurrence patterns. In cervical disease, that balance is even less stable.

Response assessment remains imperfect. Bite-on-bite biopsies improve detection of residual locoregional disease. PET/CT is more useful for interval metastases than for proving local clearance [41,42]. ctDNA adds another layer, not a final answer. Persistent detectability after induction is unfavorable [66,68]. Clearance is harder to interpret. Across neoadjuvant immunotherapy cohorts, specificity for pathologic complete response remains modest [65]. That is not good enough for binary decision-making. In this framework, ctDNA is a modifier. It should sharpen discussion, not replace it.

Timing matters as well, although the cervical evidence is thin. NeoRes II suggests worse outcomes when surgery drifts beyond ten weeks after chemoradiotherapy in incomplete responders [80]. STS/ASTRO guidance favors surgery within seven to eight weeks when the patient is ready [81]. A four-to-six-week window after induction remains a reasonable working assumption for cervical disease, especially when resection is being considered for residual tumor rather than deferred after prolonged observation. Better data are needed. For now, waiting without a clear reason is hard to justify.

Postoperative management deserves equal attention. Patients with ypT+/ypN+ disease after preoperative chemoradiotherapy and R0 resection should be evaluated for adjuvant nivolumab under current guidelines [8]. That step is often missing from older institutional algorithms because the evidence came later. It should no longer be treated as optional background context when surgery follows chemoradiotherapy. The same certainty does not apply after PD-1-based induction. Repeating or extending checkpoint blockade after prior PD-1 exposure may be reasonable in trials or selected MDT decisions, but its incremental benefit in cervical ESCC is unproven. The decision carries particular weight in cervical disease, where recurrence can be difficult to salvage and perioperative recovery is already demanding.

The functional side of the argument is easy to underweight if survival is the only endpoint in view. It should not be. Voice, swallowing, aspiration risk, prolonged recovery, and feeding-tube dependence all matter here. Organ preservation by chemoradiotherapy is real, but so is late dysphagia. Surgery may improve local control in selected patients, but it can do so at a substantial physiologic and functional cost. That tradeoff is not secondary. It is part of the treatment effect.

This review has obvious limits. The cervical evidence base is sparse. No randomized study addresses this question directly. Much of the recent enthusiasm around chemoimmunotherapy comes from thoracic cohorts, not from the cervical subsite itself. We tried to keep those boundaries visible throughout. Cervical-specific studies were prioritized. Thoracic data were used when they helped frame response depth, timing, biomarkers, or postoperative strategy, and labeled as extrapolative when that is what they were. The framework should be read in the same way. It is not a validated instrument. It is a structured way to approach a problem that is already being decided in clinic, often without structure.

### Strengths and Limitations

The principal strengths of this review are a PRISMA 2020 compliant, prospectively registered protocol; a cervical-specific synthesis built on 20 cervical-direct studies, with thoracic and meta-analytic evidence used only as labeled supporting data under a prespecified evidence-to-decision hierarchy; dual independent screening, extraction, and risk-of-bias assessment with NOS, ROBINS-I, and GRADE; and a transparent, conceptually anchored MDT framework that integrates response depth, post-induction stage, larynx-preservation feasibility, sarcopenia, ctDNA, and PD-L1 within a single decision structure.

The principal limitations are equally clear. Cervical-specific evidence is predominantly retrospective, single-center, and prone to selection bias; no randomized trial has directly addressed surgery versus continued chemoradiotherapy in this subsite. Many of the biological and response benchmarks used to anchor the framework are extrapolated from thoracic ESCC cohorts and therefore carry indirect applicability. Reported survival differences between surgical and non-surgical pathways may reflect treatment effect, selection, or both, and immortal-time bias cannot be excluded for studies in which surgery follows a fitness-dependent interval after induction. The proposed MDT selection framework has not been prospectively validated and the domain weights are consensus- and evidence-informed rather than statistically optimized; prospective, ideally multi-institutional validation against survival, functional, and quality-of-life endpoints is required before any element of the framework can be used as a clinical decision tool.

## 10. Future Directions and Research Priorities

Optimal induction regimen. Comparative trials of chemoimmunotherapy versus chemoradiotherapy in cervical esophageal cancer are needed to define optimal induction strategy, response rates, and functional outcomes. The ongoing TNT-ESCC trial (NCT06764355; [82]) evaluates a total neoadjuvant therapy approach comprising induction immunochemotherapy (tislelizumab plus paclitaxel plus cisplatin, 2 cycles) followed by chemoradiotherapy (45 Gy) and then surgery in locally advanced thoracic esophageal squamous cell carcinoma, with pathologic complete response as the primary endpoint. Results may be informative for cervical disease, but direct extrapolation will remain limited.

Surgical timing. The optimal interval between completion of induction therapy and surgery is undefined for the cervical subsite. NeoRes II data suggest a trend toward worse survival with intervals exceeding ten weeks, concentrated in incomplete responders [80]. Prospective cervical-specific studies evaluating early (4–6 weeks) versus standard (7–10 weeks) intervals are needed.

Predictive biomarkers. PD-L1 expression, tumor mutational burden, and ctDNA dynamics may predict immunotherapy response and guide selection, but none has been validated in the cervical subsite. The prognostic nutritional index, derived from serum albumin and total lymphocyte count, predicts pathologic complete response to neoadjuvant immunochemotherapy in thoracic esophageal squamous cell carcinoma (AUC 0.720) and may serve as a simple pretreatment modifier pending cervical-specific validation [83]. Composite inflammatory-nutritional indices may further refine prediction, but cervical-specific validation is lacking. A schematic of the recommended biomarker assessment windows across the pretreatment, mid-induction, post-induction, and postoperative phases is provided in Supplementary Materials Figure S1.

ctDNA-guided organ preservation. Combining TP53-centric ctDNA with PET/CT achieves AUCs of 0.80–0.86 for pathologic complete response prediction after neoadjuvant immunochemotherapy [69]. If ctDNA negativity combined with clinical complete response can reliably identify patients without viable residual tumor, organ preservation could be extended to a broader cervical population. The low specificity of ctDNA clearance for pCR (0.53) must be resolved before such a strategy is implemented [65].

Response-assessment accuracy. preSANO and preSINO inform residual disease detection in oesophageal squamous cell carcinoma, but neither resolves the cervical subsite, where fibrosis, airway proximity, and sampling limitations have greater practical consequence [41,42].

Larynx preservation criteria. Objective criteria for feasibility assessment, margin adequacy, functional outcomes, and oncologic safety require standardization. Multi-institutional prospective series with validated functional instruments are needed.

Surveillance versus surgery in complete responders. SANO-informed active surveillance needs cervical-specific study. The 48% locoregional regrowth rate in SANO, limited salvage anatomy at the cervical level, and absence of cervical-specific quality-of-life data from surveillance studies all represent critical gaps [39].

Quality of life and functional outcomes. Longitudinal assessment of voice, swallowing, vasopressor-requiring events, and global quality of life using validated instruments is essential.

Salvage strategies. The Miyata et al. [75] data (5-year OS 28–42% in 227 salvage esophagectomies, complication rate ∼49%) define the current benchmark for locoregional failure after definitive chemoradiotherapy.

## 11. Conclusions

Cervical esophageal cancer remains a low-evidence disease managed with high-stakes decisions. Definitive chemoradiotherapy is still the standard pathway for most patients. The available data nevertheless support a narrower claim: after induction therapy, surgery has a role in selected incomplete responders with resectable residual disease, acceptable functional tradeoffs, and access to experienced centers. Chemoimmunotherapy has changed the response landscape, but cervical-specific prospective data remain limited. The framework proposed here is meant to discipline selection, not to settle it. Prospective validation is required before routine clinical use.

## Data Availability

All data analyzed in this review were extracted from published studies cited in the manuscript and Supplementary Materials. The structured extraction workbook and screening log are available from the corresponding author on reasonable request.

## Supplementary Materials

The following supporting information can be downloaded at: https://www.mdpi.com/article/10.3390/cancers18111736/s1, Table S1: Search strategies by database; Table S2: Full-text exclusions with reasons (PRISMA 2020 Item 16a–b); Table S3: Supporting evidence—cervicothoracic and upper esophageal studies cited for indirect comparison (not counted as included); Table S4: Supporting evidence—thoracic contextual studies anchoring induction selection (not counted as included); Table S5: Newcastle–Ottawa Scale quality assessment; Table S6: ROBINS-I risk-of-bias assessment for non-randomized comparative studies; Table S7: Cochrane RoB 2 assessment for ESCORT-NEO/NCCES01 [14], the single phase 3 randomized trial in ESCC informing indirect evidence; Table S8: GRADE certainty of evidence; Table S9: Evidence-to-decision weighting hierarchy for direct and indirect evidence; Table S10: Source data for the survival forest plot; Table S11: Evidence basis for the functional outcomes heatmap; Figure S1: Biomarker assessment timeline.

## Abbreviations

The following abbreviations are used in this manuscript:

AE	Adverse event
CEC	Cervical esophageal cancer
ChemoIO	Chemoimmunotherapy
CI	Confidence interval
CPS	Combined positive score
CR	Complete response
CROSS	Chemoradiotherapy for Oesophageal Cancer Followed by Surgery Study
CRT	Chemoradiotherapy
CSS	Cancer-specific survival
CT	Computed tomography
ctDNA	Circulating tumor DNA
dCRT	Definitive chemoradiotherapy
DFS	Disease-free survival
ECOG	Eastern Cooperative Oncology Group
EFS	Event-free survival
E-PASS CRS	Estimation of Physiologic Ability and Surgical Stress Comprehensive Risk Score
ESCC	Esophageal squamous cell carcinoma
ESCORT-NEO	ESCORT-Neoadjuvant Oncology
GRADE	Grading of Recommendations, Assessment, Development and Evaluation
HR	Hazard ratio
IO	Immunotherapy
LA-ESCC	Locally advanced esophageal squamous cell carcinoma
LP	Larynx-preserving
LVI	Lymphovascular invasion
MDT	Multidisciplinary team
mOS	Median overall survival
MPR	Major pathologic response
N/A	Not applicable
NCDB	National Cancer Database
NCCN	National Comprehensive Cancer Network
NCIT	Neoadjuvant chemoimmunotherapy
NCRT	Neoadjuvant chemoradiotherapy
NEO-EC-01	Neoadjuvant immunochemoradiotherapy in esophageal cancer 01
NOS	Newcastle–Ottawa Scale
NR	Not reported
NS	Not significant
OS	Overall survival
pCR	Pathologic complete response
PD-1	Programmed death 1
PD-L1	Programmed death-ligand 1
PET/CT	Positron-emission tomography/computed tomography
PFS	Progression-free survival
PRISMA	Preferred Reporting Items for Systematic Reviews and Meta-Analyses
PROSPERO	International Prospective Register of Systematic Reviews
PSM	Propensity-score matching
R0	Margin-negative resection
RCT	Randomized controlled trial
RoB 2	Cochrane Risk of Bias 2
ROBINS-I	Risk Of Bias In Non-randomised Studies of Interventions
RT	Radiotherapy
SANO	Surgery As Needed for Oesophageal cancer
SCENIC	Stratified Treatment of Localized Cervical Esophageal Squamous Cell Carcinoma Induced by Neoadjuvant Immunotherapy Plus Chemotherapy
SEER	Surveillance, Epidemiology, and End Results
SMI	Skeletal muscle index
TAP	Tumor area positivity
TP	Paclitaxel and cisplatin
TPLE	Total pharyngolaryngoesophagectomy
