# Supplementary material for: Surgery After Induction Therapy for Cervical Esophageal Cancer: A Systematic Review and Proposed Multidisciplinary Selection Framework
Source: Cancers (Basel). 2026 May 26;18(11):1736. doi: 10.3390/cancers18111736 (PMC13255703; doi:10.3390/cancers18111736)
Supplement: Supplementary file 1 [file cancers-18-01736-s001.zip › Supplementary_cancers-4326907.pdf]

Supplementary Materials

Surgery After Induction Therapy for Cervical Esophageal Cancer: A Systematic Review and Proposed Multidisciplinary Selection Framework

This supplement contains the database search strategies (Table S1), the audit of full-text exclusions with reasons (Table S2), two tables of supporting evidence cited for indirect comparison but not counted as included (Tables S3 and S4), the Newcastle–Ottawa Scale quality assessment for retrospective comparative studies (Table S5), the ROBINS-I risk-of-bias assessment for prospective non-randomized studies (Table S6), the Cochrane RoB 2 assessment for ESCORT-NEO/NCCES01 (Qin 2024) (Table S7), the GRADE certainty-of- evidence summary for key outcomes (Table S8), the evidence-to-decision weighting hierarchy used to interpret direct and indirect evidence (Table S9), source data underlying the descriptive survival forest plot (Table S10), and the evidence basis underpinning the functional outcomes heatmap (Table S11). Figure S1 shows the biomarker assessment timeline.

Supplementary Table S1. Search Strategies by Database

Table S1: Database search strategies used in the review (database inception through April 2026).

Database	Search strategy
PubMed/MEDLINE	("cervical esophageal cancer"[Title/Abstract] OR "cervical esophageal carcinoma"[Title/Abstract] OR "proximal esophageal cancer"[Title/Abstract]) AND ("induction therapy"[Title/Abstract] OR "neoadjuvant therapy"[Title/Abstract] OR "chemoradiotherapy"[Title/Abstract] OR "chemoimmunotherapy"[Title/Abstract] OR "immunochemotherapy"[Title/Abstract]) AND ("surgery"[Title/Abstract] OR "esophagectomy"[Title/Abstract] OR "larynx-preserving"[Title/Abstract])
Web of Science	TS=("cervical esophageal cancer" OR "cervical esophageal carcinoma" OR "proximal esophageal cancer") AND TS=("induction therapy" OR "neoadjuvant" OR "chemoradiotherapy" OR "chemoimmunotherapy") AND TS=("surgery" OR "esophagectomy" OR "surgical resection")
Scopus	TITLE-ABS-KEY("cervical esophageal cancer" OR "cervical esophageal carcinoma" OR "proximal esophageal cancer") AND TITLE-ABS-KEY("induction therapy" OR "neoadjuvant" OR "chemoradiotherapy" OR "chemoimmunotherapy" OR "immunotherapy") AND TITLE-ABS-KEY("surgery" OR "esophagectomy" OR "larynx-preserving")
Cochrane Library	("cervical esophageal cancer" OR "proximal esophageal cancer") AND ("induction" OR "neoadjuvant" OR "chemoradiotherapy" OR "immunotherapy") AND ("surgery" OR "esophagectomy")
Manual screening	Reference lists of eligible studies, relevant reviews, and major guideline documents were screened for additional cervical-specific or clinically informative reports.

Supplementary Table S2. Full-Text Exclusions with Reasons

Eighty-seven reports were assessed at the full-text stage; 67 were excluded and 20 cervical-direct studies were retained. The exclusion categories follow the PRISMA 2020 flow diagram (main manuscript Figure 1): wrong population (n = 66) and mixed cohort (n = 1). The first panel below summarizes the aggregate count; the second panel lists named studies that a reader might expect to see among the primary-included set and explains, on a per-study basis, why each was excluded from the included set. Thoracic and meta-analytic sources listed as supporting evidence (Supplementary Tables S3 and S4) are cited for indirect comparison and are not considered full-text exclusions; they were ineligible for the primary-included set on the same population criterion but are retained in the review for biological and quantitative benchmarking.

Panel A. Full-text exclusion counts by reason (PRISMA Item 16a).

Exclusion reason	n	Operational definition
Wrong population	66	Disease not cervical esophageal. Includes thoracic ESCC, gastroesophageal junction tumors, hypopharyngeal squamous cell carcinoma, proximal-only series without a cervical-direct subset, and non-squamous histologies.
Mixed cohort	1	Study included cervical esophageal patients alongside thoracic or hypopharyngeal patients, but cervical-direct outcomes were not separable in the publication and the authors' contact did not yield an extractable subset.
**Total excluded at full-text**	**67**	

Panel B. Named full-text exclusions that may appear to meet criteria (PRISMA Item 16b).

Studies here were retrieved in full text, reviewed independently by two reviewers (I.M. and R.Z.), and excluded on the ground specified. Several of these studies are cited in the main manuscript as supporting evidence (Supplementary Tables S3 and S4); that citation does not promote them into the primary-included set.

Study	Source / design	Exclusion reason	Rationale
Wang 2026 (neoCHANCE-1)	*J Otolaryngol Head Neck Surg*; phase 2 prospective	Wrong population	Locally advanced hypopharyngeal squamous cell carcinoma with neoadjuvant tislelizumab plus afatinib. Hypopharyngeal rather than cervical esophageal disease; no cervical esophageal subset reported.
Gui 2023	*J Clin Oncol* (abstract); phase 2	Wrong population	Locally advanced hypopharyngeal SCC; tislelizumab plus chemotherapy then chemoradiotherapy or surgery. Organ-preservation question overlaps with the present review but the population is hypopharyngeal, not cervical esophageal.
Chen 2022	*Cancers*; retrospective, 14 yr	Wrong population	Postcricoid carcinoma series ($n = 72$). Postcricoid disease straddles the hypopharynx–cervical esophagus boundary; the published cohort is hypopharyngeal by site coding and was not extractable as cervical esophageal.
Guo 2025	*JAMA Surg*; multicentre prospective registry with PSM	Wrong population	Thoracic locally advanced ESCC comparing neoadjuvant chemoimmunotherapy with neoadjuvant chemoradiotherapy; cervical tumours excluded at the trial level. Cited as supporting evidence (Supplementary Table S3).
Qin 2024 (ESCORT-NEO/NCCES01)	*Nat Med*; open-label phase 3 RCT	Wrong population	Resectable thoracic locally advanced ESCC; does not enroll cervical esophageal patients. Retained as supporting evidence for indirect comparison (Supplementary Tables S7 and S4).
Zhang 2025	Prospective cohort with PSM; thoracic ESCC	Wrong population	Thoracic ESCC only. Cited as supporting evidence and assessed with ROBINS-I (Supplementary Tables S6 and S4).

Table S2 – continued from previous page

**Study**	**Source / design**	**Exclusion reason**	**Rationale**
Tian 2024	Multicentre retrospective; thoracic ESCC	Wrong population	Thoracic ESCC comparator for NCIT vs NCRT. Cited in Supplementary Table S4 for indirect context.
Shao 2025	Retrospective cohort; thoracic ESCC	Wrong population	Real-world chemo+IO induction in thoracic disease. Cited as supporting evidence in Supplementary Table S4.
Chen 2025 (thoracic)	Retrospective cohort; thoracic ESCC	Wrong population	Separate Chinese thoracic cohort replicating the pCR signal from Qin 2024. Cited as supporting evidence in Supplementary Table S4.
Yu 2024	Retrospective cohort; thoracic ESCC	Wrong population	Peri-operative safety data for thoracic chemo+IO. Cited in Supplementary Table S4.
Yan 2026 (NEO-EC-01)	Multicentre real-world; thoracic-dominant	Wrong population	Neoadjuvant immunochemoradiotherapy; cervical tumours excluded per protocol. Cited as supporting evidence in Supplementary Table S4.
Liu 2025	Retrospective cohort; thoracic ESCC	Wrong population	pCR benchmark in thoracic squamous disease after chemo+IO. Cited in Supplementary Table S4.
Xu 2022 (thoracic cohort)	Retrospective cohort; thoracic ESCC	Wrong population	Thoracic cohort distinct from the cervical SEER analysis cited as Xu 2022 in the main manuscript. Cited as supporting evidence in Supplementary Table S4.
Representative mixed-cohort exclusion	Retrospective single-centre series	Mixed cohort	Cervical and upper-thoracic patients reported jointly without a separable cervical subset; author contact did not yield an extractable cervical-only dataset.

Sixty additional full-text reports were excluded on the same population ground (non-cervical esophageal disease, hypopharyngeal SCC, non-squamous histology, case reports, editorials, duplicates of included analyses, or cohorts without extractable cervical-direct outcomes). The complete list with individual exclusion reasons is maintained in the structured screening log and is available from the corresponding author on reasonable request.

Note. Panel A matches the numeric counts shown in the PRISMA 2020 flow diagram (Figure 1). Panel B follows PRISMA 2020 Item 16b by citing and explaining the exclusion of studies most likely to be expected among the included set. Screening was dual-reviewer; disagreements were adjudicated by a third reviewer (O.C.).

Supplementary Table S3. Supporting Evidence: Cervicothoracic and Upper Esophageal Studies

The studies in this table cover upper-thoracic or proximal-esophageal disease in which cervical outcomes are not the exclusive focus. They are cited in the review for indirect comparison, particularly for larynx-preservation feasibility, preoperative planning, and induction sequencing at the thoracic inlet. They are not counted in the primary-included set, which is restricted to cervical-direct studies (main manuscript Section 6). Full per-study variables sit in the extraction workbook that accompanies this review.

Study	Design	Population focus	Cervical relevance
Guo et al., 2025	Multicentre prospective registry with PSM	Thoracic LA-ESCC (N = 1,428; PSM 532+532)	Largest comparative NCIT vs. NCRT signal; 2-yr OS 81.3% vs. 71.3%. Extrapolable direction for upper-cervical disease but excludes cervical tumours.
Huang et al., 2022	Single-centre retrospective	Upper-thoracic ESCC	Surgical feasibility and LP benchmarks at the upper thoracic level; informs anatomic continuity for cervical LP planning.
Yu et al., 2022	Retrospective cohort	Thoracic ESCC with cervical adjacency	Supports induction-to-surgery sequencing at the thoracic inlet.
Zeng et al., 2025	Retrospective cohort	Thoracic ESCC with cervical-adjacent anatomy	Contextualizes dose–response for cervical planning in mixed cohorts.
Makino et al., 2016	Single-centre consecutive series	Cervical and cervicothoracic (LP intent, N = 100)	Cervical-direct primary-included study, listed again here only as the cervicothoracic anchor for preoperative dose calibration (60 Gy vs. 40 Gy); it is not double-counted in the included set.

Abbreviations: LA-ESCC, locally advanced esophageal squamous cell carcinoma; LP, larynx preservation; NCIT, neoadjuvant chemoimmunotherapy; NCRT, neoadjuvant chemoradiotherapy; OS, overall survival; PSM, propensity-score matching.

Supplementary Table S4. Supporting Evidence: Thoracic Contextual Studies Anchoring Induction Selection

The studies listed in this table are thoracic-dominant reports that anchor indirect evidence for chemoimmunotherapy as an induction option and for comparative survival in ESCC outside the cervical subsite. They are cited in the main manuscript (Sections 3 and 8) for biological rationale and dose-intensity context; none of them provide cervical-direct outcomes and they are therefore supporting rather than primary-included evidence.

Study	Design	Comparator/Arm	Contextual role for cervical synthesis
Qin et al., 2024 (ESCORT-NEO)	Phase 3 RCT, three-arm	Cam+nab-TP vs. Cam+TP vs. TP	Single phase 3 RCT of neoadjuvant chemoimmunotherapy in ESCC; the pCR difference between Cam+nab-TP and TP (28.0% vs. 4.7%) is the quantitative anchor for indirect cervical extrapolation. RoB 2 appears in Supplementary Table S7.
Tian et al., 2024	Multicentre retrospective	NCIT vs. NCRT, thoracic ESCC	Supports comparable or superior pathologic response with lower postoperative complication signal.
Shao et al., 2025	Retrospective cohort	Chemo+IO induction, thoracic	Real-world pCR and safety profile consistent with Qin et al.
Chen et al., 2025	Retrospective cohort	Chemo+IO induction, thoracic ESCC	Replicates pCR signal in a separate Chinese cohort.

Table S4 – continued from previous page

Study	Design	Comparator/Arm	Contextual role for cervical synthesis
Yu et al., 2024	Retrospective cohort	Induction chemo+IO, thoracic	Adds peri-operative safety data relevant to the cervical surgical pathway.
Yan et al., 2026 (NEO-EC-01)	Multicentre real-world	Neoadjuvant immunochemoradio-therapy	Confirms feasibility of trimodality intensification; excluded cervical but directionally supportive.
Liu et al., 2025	Retrospective cohort	Chemo+IO induction	Reports pCR of 50% in squamous histology; magnitude consistent with ESCORT-NEO.
Xu et al., 2022 (thoracic)	Retrospective cohort	Comparative induction strategies, thoracic	Contextual comparator distinct from the cervical SEER analysis cited in the main manuscript (Xu et al., 2022, cervical SEER cohort).

Abbreviations: Cam, camrelizumab; Chemo+IO, chemotherapy plus immune-checkpoint inhibitor; nab-TP, albumin-bound paclitaxel and cisplatin; NCIT, neoadjuvant chemoimmunotherapy; NCRT, neoadjuvant chemoradiotherapy; pCR, pathologic complete response; RoB, risk of bias; TP, paclitaxel and cisplatin.

Note on evidence use. The primary-included set of this review is limited to cervical-direct studies (main manuscript Table 1, Sections A, B, and E; n = 20). Cervicothoracic and upper-esophageal sources (Supplementary Table S3) and thoracic- dominant sources (this table) are cited for indirect comparison and biological rationale; they are not counted in the included set. The complete per-study extraction covering population, intervention, outcomes, and risk-of-bias variables is maintained in the structured extraction workbook that accompanies this review.

Supplementary Table S5. Risk of Bias Assessment: Newcastle–Ottawa Scale (NOS)

The Newcastle–Ottawa Scale (NOS) was applied to comparative cohort and registry-based studies included in the review. Items are rated across three domains: Selection (maximum 4 stars), Comparability (maximum 2 stars), and Outcome (maximum 3 stars); the maximum possible score is 9 stars. Studies scoring ≥ 7 were considered at low risk of bias; 5–6 at moderate risk; ≤ 4 at high risk. Single-arm surgical series, prospective phase II trials without a comparator group, and meta-analyses were excluded from NOS scoring as the instrument was not designed for these designs; their methodological limitations are addressed in the narrative text. Prospective non-randomized comparative studies were assessed separately using ROBINS-I (Supplementary Table S6).

Table S5: Newcastle–Ottawa Scale assessment. S = Selection (max 4); C = Comparability (max 2); O = Outcome (max 3); NOS = total (max 9). Studies scoring ≥7: low risk of bias; 5–6: moderate; ≤4: high.

**Study**	**Design**	**S**	**C**	**O**	**NOS**	**Principal limitations**
Miyakoshi 2022	Single-center retrospective cohort	3	1	3	7	No PSM; treatment allocation driven by response and anatomy; heterogeneous induction (chemotherapy subset); single institution.
Valmasoni 2018	Retrospective 3-arm cohort	3	1	3	7	Non-randomized; surgical arm enriched for non-CRs; no formal matching; institutional selection bias likely.
Takebayashi 2017	Retrospective comparative	2	1	3	6	Small sample ($N = 49$); no PSM or multivariable adjustment; CRT arm had salvage surgery crossover; primary treatment comparison, not induction-plus-surgery pathway.
Sabbagh 2023	NCDB retrospective	3	1	3	7	Mixed cervical and upper-third population; no response data in registry; cervical-specific subgroup showed no independent benefit; NCDB lacks functional outcome data.
Alhalabi 2025	NCDB retrospective, PSM	4	2	3	9	Benefit driven by thoracic tumors; cervical subgroup not independently significant; registry lacks induction response and functional data; PSM cannot adjust for unmeasured confounders.
Patel 2023	NCDB retrospective	4	1	3	8	Primary comparison is any definitive vs no treatment; surgery did not achieve statistically significant OS advantage over CRT in adjusted analyses; R0/pCR available in surgical subgroup only.

Study	Design	S	C	O	NOS	Principal limitations
Qi 2020	SEER retrospective, PSM	4	2	3	9	Registry lacks response data and functional outcomes; HR 0.75 did not reach significance ($p = 0.06$); PSM limited to available covariates.
Xu 2022	SEER retrospective, PSM	4	2	3	9	Large population-based sample; registry constraints apply (no response, no functional data); 10-year OS endpoint susceptible to lead-time bias in registry setting.
Yuan 2025	Retrospective cohort, LP vs TPLE	3	1	3	7	Allocation to LP vs TPLE driven by tumor anatomy, not random; TPLE group had intrinsically more advanced disease; R0 rates systematically lower in LP arm; 2-year endpoint only.
Zhang 2025	Prospective cohort, PSM	4	2	3	9	Thoracic ESCC only; prospective ascertainment strengthens internal validity; institutional preference for Chemo+IO arm; cervical applicability indirect.

Abbreviations: CRT, chemoradiotherapy; CR, complete response; LP, larynx-preserving; NCDB, National Cancer Database; NOS, Newcastle–Ottawa Scale; OS, overall survival; PSM, propensity-score matching; SEER, Surveillance, Epidemiology, and End Results; TPLE, total pharyngolaryngoesophagectomy.

Supplementary Table S6. ROBINS-I Risk-of-Bias Assessment: Prospective Non-Randomized Studies

The Risk Of Bias In Non-randomised Studies – of Interventions (ROBINS-I) tool was applied to prospective non-randomized comparative studies included in the review. ROBINS-I assesses bias across seven domains: (1) confounding, (2) selection of participants, (3) classification of interventions, (4) deviations from intended interventions, (5) missing data, (6) measurement of outcomes, and (7) selection of the reported result. Judgements for each domain are: Low, Moderate, Serious, Critical, or No information. The overall ROBINS-I judgement reflects the most serious domain rating. Retrospective cohort and registry studies were assessed using the Newcastle–Ottawa Scale (Supplementary Table S5). Single-arm series and meta-analyses are not included in either RoB tool assessment.

Table S6: ROBINS-I assessment for prospective non-randomized comparative studies. D1–D7 = domains 1–7 as listed above. L = Low; M = Moderate; S = Serious; NI = No information.

Study	D1 Confound.	D2 Select.	D3 Classif.	D4 Deviat.	D5 Missing	D6 Outcome	D7 Report.	Overall
Zhang 2025 (prospective cohort, PSM; thoracic ESCC; Chemo+IO vs upfront Sx)	M	L	L	M	L	L	L	**Moderate**

Domain notes. **D1 (Confounding):** Propensity-score matching was performed; however, the Chemo+IO arm reflects institutional preference and potential selection of patients considered more likely to benefit, introducing residual confounding not addressable by PSM alone. **D4 (Deviations):** No formal adherence protocol reported; treatment crossover or modification could not be fully excluded in the prospective registry period. All other domains were judged Low risk.

Reference: Sterne JAC, Hernán MA, Reeves BC, et al. ROBINS-I: a tool for assessing risk of bias in non-randomised studies of interventions. *BMJ.* 2016;355:i4919. doi:10.1136/bmj.i4919

Supplementary Table S7. Cochrane RoB 2 Assessment: Qin et al., 2024 (ESCORT-NEO/NCCES01)

Qin 2024 is the single phase 3 randomized trial of neoadjuvant chemoimmunotherapy in ESCC currently available. It enrolled thoracic locally advanced disease rather than a cervical-direct population, which places the trial in the supporting rather than the primary-included set for this review; we apply Cochrane RoB 2 because the design is randomized and the tool is domain-appropriate. The study is a multicenter, open-label, three-arm comparison of camrelizumab plus albumin-bound paclitaxel and cisplatin (Cam+nab-TP), camrelizumab plus paclitaxel and cisplatin (Cam+TP), and chemotherapy alone (TP), with pathologic complete response on the resection specimen as the primary endpoint (n = 391).

Domain	Signalling judgement	Rating
D1. Randomization process	Centralized randomization with stratification and allocation concealment; no meaningful baseline imbalance reported between arms.	Low
D2. Deviations from intended interventions (assignment effect)	Open-label design is unavoidable given the biologic vs. chemotherapy regimens. Pathologic endpoint mitigates performance bias; adjuvant camrelizumab per protocol could influence downstream outcomes but does not affect the primary pCR analysis window.	Some concerns
D3. Missing outcome data	Primary pCR evaluable in the surgical cohort; per-protocol and intention-to-treat analyses are reported; attrition from randomization to surgery is balanced across arms.	Low
D4. Measurement of the outcome	pCR assessed on resection specimens by blinded pathology review per predefined tumor regression grading; objective endpoint.	Low

Table S7 – continued from previous page

Domain	Signalling judgement	Rating
D5. Selection of the reported result	Pre-specified primary and key secondary endpoints match the published analysis; registration number consistent between protocol and publication.	Low
**Overall**	Open-label design is the principal limitation; primary endpoint is a pathology-based measure that is insensitive to performance bias.	**Some concerns**

Reference. Sterne JAC, Savović J, Page MJ, et al. RoB 2: a revised tool for assessing risk of bias in randomised trials. *BMJ.* 2019;366:l4898. doi:10.1136/bmj.l4898

Supplementary Table S8. GRADE Certainty of Evidence

Certainty of evidence was rated using the Grading of Recommendations, Assessment, Development and Evaluation (GRADE) framework for five key outcomes that directly inform the proposed selection framework. Starting certainty was Low for all outcomes because no randomized controlled trial data exist for this disease and intervention. Ratings reflect domain-specific downgrading for risk of bias, inconsistency, indirectness (predominance of thoracic data extrapolated to the cervical subsite), imprecision (small samples, wide confidence intervals), and suspected publication bias.

Table S8: GRADE certainty-of-evidence assessment. ↓ = one-level downgrade; ↓↓ = two-level downgrade. RoB = risk of bias; Incons. = inconsistency; Indir. = indirectness; Imprec. = imprecision; PubB = publication bias.

Outcome	Studies (N)	RoB	Incons.	Indir.	Imprec.	PubB	Certainty
OS: surgery vs definitive CRT in non-CR patients (cervical-specific)	4 cervical cohorts	↓↓	↓	–	↓	Suspected	**Very Low**
Larynx preservation feasibility: T1–2 cervical tumors	2 cervical series	↓	–	–	↓	Suspected	**Low**
pCR rate with neoadjuvant chemoimmunotherapy (ESCC)	3 meta-analyses + multiple cohorts	↓	↓	↓↓	–	Suspected	**Very Low** (cervical); **Low** (thoracic)
30-day surgical mortality (cervical esophagectomy)	5 cervical/proximal series	↓	–	–	↓	Suspected	**Low**
CRT-associated stricture rate vs surgery (cervical subsite)	2 cervical/hypopharyngeal series + extrapolation	↓	–	↓	↓	Suspected	**Low**

Downgrading rationale. **OS (surgery vs CRT):** all studies are retrospective with treatment allocation driven by response status, anatomy, and institutional preference (RoB ↓↓); HRs range 0.28–0.85, reflecting different populations rather than genuine inconsistency, but the heterogeneity merits downgrading (Incons. ↓); cervical-specific comparative series are small (N = 49–148), limiting precision. **Larynx preservation:** single-arm series with no randomization; R0 rates vary substantially by tumor anatomy (inherent confounding). **pCR with chemoimmunotherapy:** thoracic-dominant evidence; cervical disease is anatomically distinct in dosimetry and reconstructive constraints; SCENIC trial (N = 28) provides the only cervical-specific data, with immature follow-up. **Surgical mortality:** consistent across series but all retrospective with volume-dependent variation. **Stricture:** cervical-specific rates plausible but the dominant CRT estimate derives from a single 20-year series at one institution.

Supplementary Table S9. Evidence-to-Decision Weighting Hierarchy

This table summarizes how direct cervical evidence and indirect thoracic or biomarker evidence were weighted during narrative interpretation and framework construction. The values are interpretive weights, not statistically estimated coefficients, and were used to make the direction of evidence use transparent.

Table S9: Prespecified evidence-to-decision weighting hierarchy used for the proposed cervical ESCC selection framework.

Evidence source	Default weight	Use in framework	Safeguard against overinterpretation
Cervical-direct comparative cohorts or registry analyses with extractable cervical estimates	1.00	Primary basis for response, stage, laryngeal feasibility, and survival interpretation	Reported as observational associations; no causal claim made without prospective validation
Cervical-direct single-arm surgical or CRT series	0.75	Functional feasibility, morbidity, larynx preservation, pCR range, and operative safety	Used descriptively; not used to infer comparative effectiveness
Mixed cervicothoracic or upper-esophageal datasets with cervical subgroups or anatomically relevant upper-third estimates	0.50	Supportive context for survival and treatment sequencing when cervical-only estimates are sparse	Downgraded for indirectness and checked against cervical-direct signals
Thoracic ESCC randomized trials, prospective cohorts, or thoracic-dominant meta-analyses	0.25–0.50	Biologic rationale, chemoimmunotherapy response benchmarks, timing, and postoperative strategy	Labeled as extrapolative; not counted as primary included cervical evidence

Evidence source	Default weight	Use in framework	Safeguard against overinterpretation
Cross-disease or thoracic biomarker studies of PD-L1, ctDNA, inflammatory-nutritional indices, or sarcopenia	0.25	Qualitative modifiers only	No numerical change to the core score; cervical-specific assay thresholds and net benefit remain unvalidated

Supplementary Table S10. Source Data for the Survival Forest Plot

Table S10: Study-level source data for the descriptive overall-survival forest plot.

Study	Comparison	HR	95% CI	Derivation
Miyakoshi 2022	Surgery vs definitive chemoradiotherapy in ≥T3 cervical ESCC	0.28	0.11–0.71	Reported
Valmasoni 2018	Surgery vs definitive chemoradiotherapy in non-complete responders	0.65	0.44–0.96	Approximated from Kaplan–Meier curves
Takebayashi 2017	Surgery vs definitive chemoradiotherapy in all treated patients	0.85	0.48–1.50	Approximated from Kaplan–Meier curves
Sabbagh 2023	Neoadjuvant CRT plus surgery vs definitive CRT alone	0.77	0.61–0.95	Reported; published comparator inverted
Patel 2023	Esophagectomy vs no esophagectomy within a definitive-treatment cohort	0.84	0.65–1.08	Reported; contextual only

Supplementary Table S11. Evidence Basis for the Functional Outcomes Heatmap

Table S11: Qualitative evidence map underlying the functional outcomes heatmap. This table records the directional judgments used in the figure and is not a pooled quantitative comparison.

**Domain**	**More favorable modality**	**Basis for assignment**	**Principal sources**
Acute mortality (30-day)	Definitive CRT	Early treatment-related mortality is generally lower with non-surgical treatment than after esophagectomy.	Comparative cervical and proximal series; surgical reports
Anastomotic leak	Definitive CRT not applicable	Anastomotic leak is unique to surgery.	Surgical series only
Recurrent laryngeal nerve injury	Definitive CRT	Immediate operative nerve injury is more frequent after surgery.	Makino 2016; Liu 2022
Stricture requiring dilation	Surgery	Late stricture burden is often greater after CRT, especially at the cervical or hypopharyngeal subsite.	Hamer 2019; Prisman 2013
Chronic dysphagia	Surgery	Persistent swallowing dysfunction remains a major limitation after CRT in long-term survivors.	Hamer 2019; Prisman 2013
Feeding tube dependence at 1 year	Surgery	Long-term tube dependence tends to be more common after CRT than after successful surgical recovery.	Comparative cervical and proximal series
Voice impairment (severe)	Definitive CRT	Severe voice dysfunction may follow either treatment, but operative RLN injury is a major surgical driver.	Makino 2016; Dai 2020; surgical series
Quality of life (long-term)	Slightly favors surgery in selected survivors	Evidence is limited; thoracic data report late swallowing-related deficits after CRT.	CROSS quality-of-life reports; cervical extrapolation
Salvage options after failure	Definitive CRT	Failure after CRT may still leave a surgical salvage pathway; failure after primary surgery is harder to rescue locally.	Miyata 2022
Organ preservation	Definitive CRT	Organ preservation is inherent to non-surgical management.	Cervical CRT literature
Larynx preservation when feasible	Both, slight advantage to CRT	Both modalities may preserve the larynx in selected cases, although CRT does so more naturally.	Cervical surgical and CRT series
Local disease control	Slightly favors surgery in selected non-complete responders	Surgical consolidation may improve local control in selected patients with residual disease after induction.	Valmasoni 2018; Miyakoshi 2022; Takebayashi 2017

Supplementary Figure S1. Biomarker Assessment Timeline

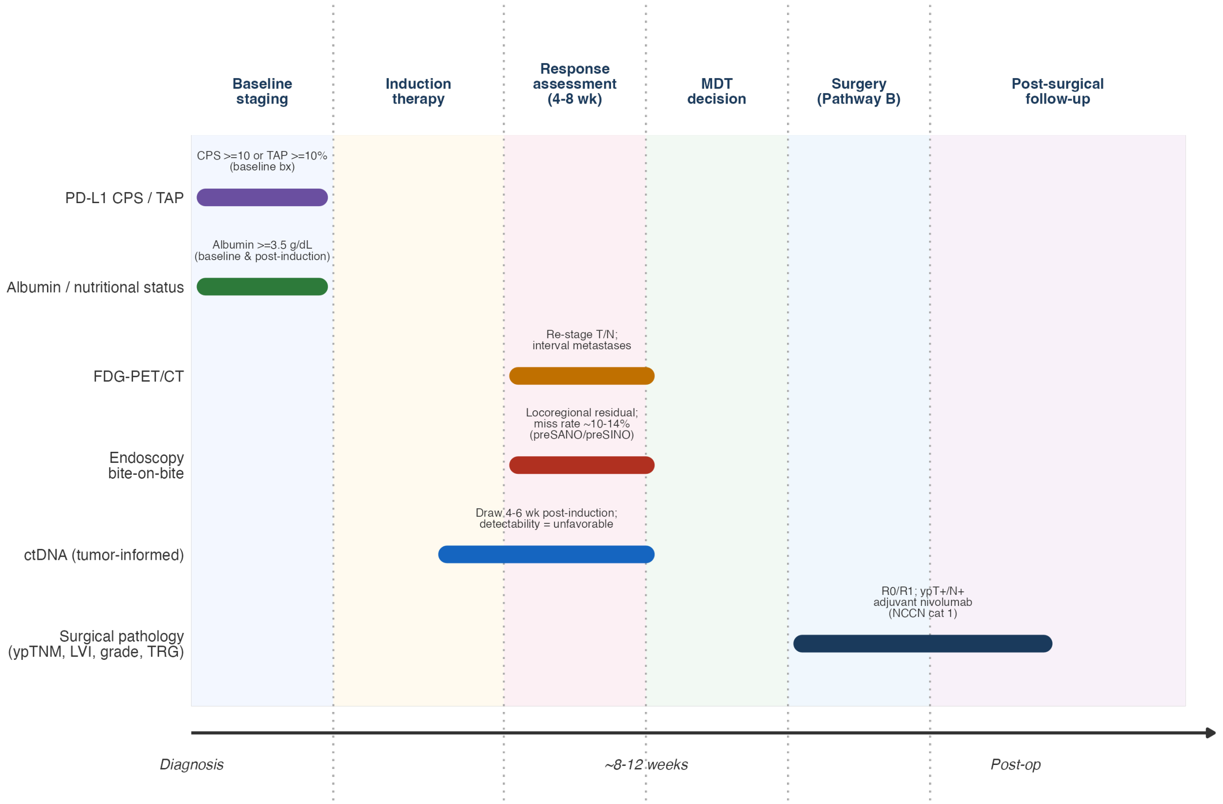

Figure S1: Biomarker assessment timeline across the induction-to-surgery pathway in cervical esophageal cancer. The schematic summarizes the timing of baseline PD-L1 assessment, nutritional evaluation, post-induction PET/CT, endoscopic bite-on-bite biopsy, ctDNA analysis, and postoperative pathology review. It is a framework summary, not a validated clinical algorithm.
